# Annexin A3 Represses Endothelial Permeability and Inflammation During Sepsis via Actin Cytoskeleton Modulation

**DOI:** 10.1002/advs.202416904

**Published:** 2025-03-28

**Authors:** Manyu Xing, Shuang Liang, Wei Cao, Qulian Guo, Wangyuan Zou

**Affiliations:** ^1^ Department of Anesthesiology Xiangya Hospital Central South University Changsha 410008 China; ^2^ National Clinical Research Center for Geriatric Disorders Xiangya Hospital Changsha 410008 China

**Keywords:** activating transcription factor 2, annexin A3, cell permeability, endothelial cell, sepsis

## Abstract

Increased endothelial permeability and a dysregulated inflammatory response play key roles in organ damage in sepsis. The role of annexin A3 (ANXA3) in regulating endothelial permeability and inflammation during sepsis is explored using ANXA3 knockout mice and primary human umbilical vein endothelial cells (HUVECs). The absence of ANXA3 exacerbated sepsis outcomes, including increased mortality, lung injury, leukocyte infiltration, and vascular permeability. ANXA3 is highly expressed in endothelial cells and its loss results in the formation of cytoskeletal stress fibers and a decrease in the expression of the junction proteins zonula occludens (Zo)‐1, vascular endothelial (VE)‐cadherin, and claudin 5, leading to increase permeability. ANXA3 knockdown also upregulates E‐selectin (CD62E) expression through the phosphorylation of activating transcription factor 2 (ATF2), which increases monocyte adhesion in HUVECs after LPS stimulation. Inhibiting actin polymerization reverse these effects. Thus, ANXA3 stabilizes the actin cytoskeleton, playing a protective role in endothelial dysfunction during sepsis.

## Graphical abstract

Annexin A3 (ANXA3) plays an endogenous protective role in endothelial dysfunction in sepsis by stabilizing the actin cytoskeleton, modulating cell permeability, and inhibiting pATF2/CD62E inflammatory signaling.

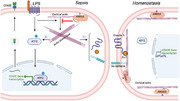

## 1 Introduction

Sepsis is a complex disease state resulting from a dysregulated host response to infection,^[^ ^1^ ^]^ and the development of organ dysfunction is directly linked to the fatality rate of sepsis.^[^ ^2^ ^]^ Extensive vascular leakage and inflammation secondary to endothelial cell (EC) dysfunction are major causes of multiorgan failure in sepsis.^[^ ^3^ ^]^ Endothelial vascular leakage is mediated via the disassembly of tight junctions (TJs)^[^ ^4^ ^]^ and adherens junctions (AJs)^[^ ^5^ ^]^ which in turn is regulated by loss of zonula occludens (Zo)‐1, claudin 5, and vascular endothelial (VE)‐cadherin.^[^ ^6^ ^]^ This is further promoted by contractile forces generated by actin stress fiber formation.^[^ ^7^ ^]^ Endothelial activation during sepsis is manifested as the production of adhesion molecules E‐selectin (CD62E) and intercellular adhesion molecule‐1 (ICAM–1) et al. on the surface of ECs,^[^ ^8^ ^]^ which are essential for polymorphonuclear neutrophil (PMN) rolling, adhesion, and migration.^[^ ^9^ ^]^ The expression of these molecules dependent on the transcription factors (TFs) including nuclear factor kappa‐B (NF–κB) or activator protein‐1(AP–1).^[^ ^10^ ^]^ Importantly, actin stress fibers also play a critical role in facilitating those TFs translocation to the nucleus^[^ ^11^ ^]^ and thereby EC inflammation.^[^ ^12^ ^]^ Therefore, regulating the actin cytoskeleton to maintain or restore vascular endothelial homeostasis is a crucial determinant of the prognosis of sepsis.

The annexin A (ANXA) family of proteins includes 12 members, ANXA1–ANXA11 and ANXA13.^[^ ^13^ ^]^ These proteins share a similar structure, featuring a highly homologous core domain and a specific N‐terminal domain. ANXAs bind to phospholipid membranes in a calcium‐dependent manner and as major regulator of actin remodeling,^[^ ^14^ ^]^ participate in various processes, such as cell phagocytosis, migration, and signaling.^[^ ^15^ ^]^ ANXA1,^[^ ^16^ ^]^ ANXA2,^[^ ^17^ ^]^ and ANXA5^[^ ^18^ ^]^ were found to play critical protective roles in host defense during sepsis.^[^ ^19^ ^]^ Studies indicated that ANXAs potentially function in maintaining vascular homeostasis and EC function.^[^ ^20^ ^]^ Notably, ANXA1^[^ ^21^ ^]^ and ANXA2^[^ ^22^ ^]^ contribute to blood–brain barrier integrity via actin cytoskeleton. Additionally, ANXA2 promotes pulmonary microvascular integrity by activating VE‐cadherin‐related phosphatase, which prevents vascular leakage in hypoxic mice.^[^ ^23^ ^]^

Our group has been continuously investigating ANXA3 and after demonstrating its regulatory role in neural development^[^ ^24^ ^]^ and neuropathic pain,^[^ ^25^ ^]^ we shifted our focus to its role in sepsis. In recent years, a series of studies on ANXA3 and sepsis have been reported. Data from public datasets indicate that the abundance of ANXA3 in the blood increases markedly during sepsis.^[^ ^26^ ^]^ Liang et al. reported that ANXA3 is upregulated in lung tissue during sepsis and that depletion of ANXA3 alleviates inflammation and apoptosis in sepsis‐induced acute lung injury (ALI).^[^ ^27^ ^]^ Another study revealed that ANXA3 expression was predominantly downregulated in the lung after sepsis modeling.^[^ ^28^ ^]^ Significant progress in the understanding of ANXA3 in sepsis has been made; nevertheless, this topic is also accompanied by some controversies and requires further investigation. Further studies are needed to better understand the role of ANXA3 in sepsis. ANXA3 has also been linked to EC function, in vitro experiments revealed that it can induce the migration of ECs.^[^ ^29^ ^]^ In vivo studies have shown that the depletion of ANXA3 causes defective embryonic vascular development in Xenopus laevis and mouse.^[^ ^30^ ^]^ Collectively, these findings strongly suggest that ANXA3 may play a crucial role in the regulation of EC function.

In this study, we discovered that ANXA3 knockout mice exhibited increased pulmonary microvascular permeability, augmented leukocyte infiltration, and decreased survival rates subsequent to LPS‐induced sepsis. We found that the knockdown of ANXA3 in primary human umbilical vein endothelial cells (HUVECs) led to the formation of stress fibers in the intracellular actin cytoskeleton and thus resulted in a reduction in tight junction and adherens junction proteins, which in turn caused an increase in EC permeability. Moreover, the knockdown of ANXA3 accelerated LPS‐induced activating transcription factor 2 (ATF2) phosphorylation and CD62E expression, thereby increasing EC activation and facilitating leukocyte adhesion. Collectively, these results offer novel insights into the mechanisms through which ANXA3 regulates EC permeability and activation during sepsis.

## 2 Results

### 2.1 ANXA3 Ablation Aggravates Mortality and Exacerbates Lung Injury in Sepsis

To investigate the role of ANXA3 in sepsis, ANXA3 knockout (ANXA3^−/−^) mice were successfully constructed with CRISPR/Cas9 technology (Figure S1A, Supporting Information). PCR was performed to identify heterozygous, wild‐type (WT), and knockout mice (Figure S1B, Supporting Information). The efficiency of ANXA3 deletion was confirmed through qRT–PCR (Figure S1C, Supporting Information) and western blotting (WB) (Figure S1D,E). No noticeable differences in the appearance or anatomy of various organs were detected between ANXA3^−/−^ and WT mice (Figure S1F, Supporting Information), and ANXA3 ablation did not impact the overall growth or development of the mice (Figure S1G, Supporting Information).

**Figure 1:**
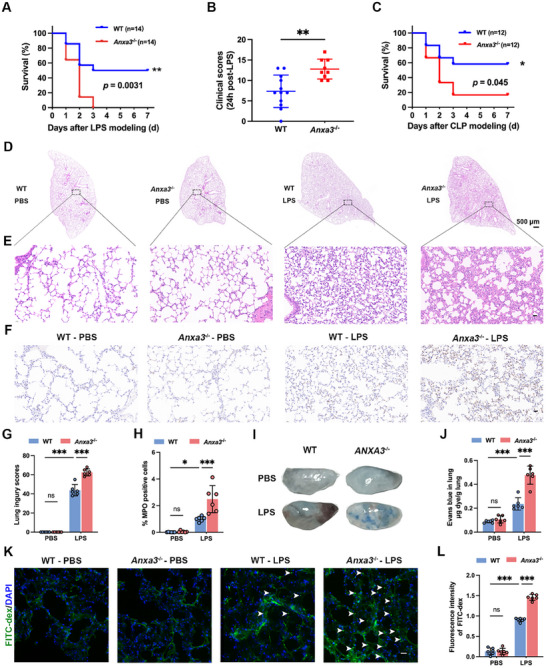
ANXA3 deficiency aggravates the response to sepsis. A) Survival analysis of male WT and ANXA3^−/−^ mice after LPS treatment (10 mg kg^−1^), *n* = 14/group. *p* = 0.031, log‐rank (Mantel–Cox) test. B) At 24 h after LPS treatment, the mice were scored for the presence or absence of six different macroscopic signs of sepsis. *n* = 9–12, ^**^ *p* < 0.01, two‐tailed unpaired Student's *t* test. C) Survival analysis after establishment of the CLP model in male WT and ANXA3^−/−^ mice, *n* = 12, *p* = 0.045, log‐rank (Mantel–Cox) test. D,E) At 18 h after LPS treatment, representative lung histological changes were assessed by H&E staining in different groups of mice. Scale bar = 500 µm in D) and scale bar = 25 µm in E). G) Lung injury scores of pulmonary damages in different groups. *n* = 6, ^***^ *p* < 0.001; two‐way ANOVA with Tukey's post hoc test. F,H) Mouse lung tissue samples from different groups were subjected to immunostaining for MPO and positive cell count analysis. Scale bar = 25 µm, *n* = 6; ^***^ *p* < 0.001; two‐way ANOVA with Tukey's post hoc test. I) Evans blue dye was injected into the mice at 18 h after LPS treatment, after which the lung tissue was harvested for imaging. J) The data were quantified as the Evans blue dye quantity (µg) per g of dry tissue weight. K) A representative photograph of FITC‐dextran leakage into the lungs (indicated by the arrows) of a mouse, as determined by the appearance of intravenously injected FITC‐dextran in vivo, is shown. The scale bar represents 20 µm. L) The relative FITC‐dextran leakage of each tissue sample was calculated from the fluorescence intensity (A.U.). ^***^ *p* < 0.001, two‐way ANOVA with Tukey's post hoc test.

Next, age‐ and weight‐matched WT and ANXA3^−/−^ mice were used to establish a lipopolysaccharide (LPS)‐mediated sepsis model. Survival analysis revealed that male ANXA3^−/−^ mice had a significantly greater mortality rate than WT mice did when challenged with a 10 mg kg^−1^ dose of LPS; 100% of ANXA3^−/−^ mice died within 72 h after LPS injection, whereas only ≈50% of WT mice died within seven days (**Figure** **1**A). The clinical behavior scores of male ANXA3^−/−^ mice were also significantly greater than those of WT mice 24 h after modeling (Figure 1B). However, mortality did not significantly differ between female WT and ANXA3^−/−^ mice after LPS injection (Figure S2A, Supporting Information), and therefore, subsequent experiments were conducted in male mice. Additionally, when the effect of a higher dose of LPS (15 mg kg^−1^) was investigated, no significant difference in mortality was detected between WT and ANXA3^−/−^ mice (Figure S2B, Supporting Information). However, compared with WT mice, ANXA3^−/−^ mice presented a more pronounced reduction in core temperature at 12 h postmodeling (Figure S2C, Supporting Information), indicating that ablation of ANXA3 exacerbates early‐stage sepsis symptoms. We also used the cecal ligation and puncture (CLP)‐mediated sepsis model and observed that male ANXA3^−/−^ mice still presented a higher mortality rate compared to WT mice (Figure 1C). Collectively, these findings strongly imply that ANXA3 plays a pivotal role in enabling effective host defense mechanisms during sepsis. To explore the effect of ANXA3 deficiency on organ damage during sepsis pathogenesis, we conducted histological analyses of the lungs, kidneys, livers, spleens, and intestines of WT and ANXA3^−/−^ mice. During sepsis, ANXA3^−/−^ mice presented more severe lung injury (Figure 1D,E,G), whereas no significant differences were observed in liver, spleen, kidney, or intestine injury (Figure S3A–D). Disruption of endothelial barrier integrity and consequent pulmonary inflammation are typical symptoms of ALI. Increased leukocyte infiltration in the lungs of ANXA3^−/−^ mice at 18 h after LPS treatment was identified by MPO immunostaining (Figure 1F–H). We further assessed lung vascular permeability by measuring Evans blue (EB) dye and FITC‐dextran leakage. The results revealed an increase in EB dye in the lung tissues of WT mice after LPS modeling; ANXA3 deletion further increased EB dye leakage (Figure 1I,J). Additionally, more fluorescent material was observed in the lung tissues of ANXA3^−/−^ mice than in those of WT mice following tail vein injection of FITC‐dextran (Figure 1K,L). These findings indicate that ANXA3 deletion exacerbates lung vascular permeability and inflammation during sepsis.

The lung endothelium is critical for ALI pathogenesis.^[^ ^3^, ^31^ ^]^ Single‐cell RNA sequencing (scRNA‐seq) showed ANXA3 is expressed predominantly in mouse ECs^[^ ^32^ ^]^ (Figure S4A, Supporting Information) and highly expressed in pulmonary vascular ECs^[^ ^33^ ^]^ (Figure S4B, Supporting Information). Then, we confirmed the typical localization of ANXA3 within CD31‐labeled ECs in mouse lung tissues (Figure S4D,E) through immunofluorescence (IF) staining analysis. Additionally, scRNA‐seq analysis of vascular ECs from lung tissue under homeostatic and inflammatory conditions (LPS stimulation)^[^ ^34^ ^]^ revealed a slight decrease in ANXA3 expression in pulmonary vascular ECs during the acute injury phase (6 and 24 h), followed by a restoration at the recovery phase (168 h) (Figure S4C, Supporting Information). We also revealed a reduction in ANXA3 in the perivascular area of the lung tissues after LPS injection (Figure S4F, Supporting Information) through immunohistochemical staining. These findings suggest that ANXA3 may play a role in EC injury and repair during sepsis.

### 2.2 Depletion of ANXA3 Decreases the Expression of TJs and AJs Proteins and Increases the Expression of Adhesion Molecules in Lung Tissue

**Figure 2:**
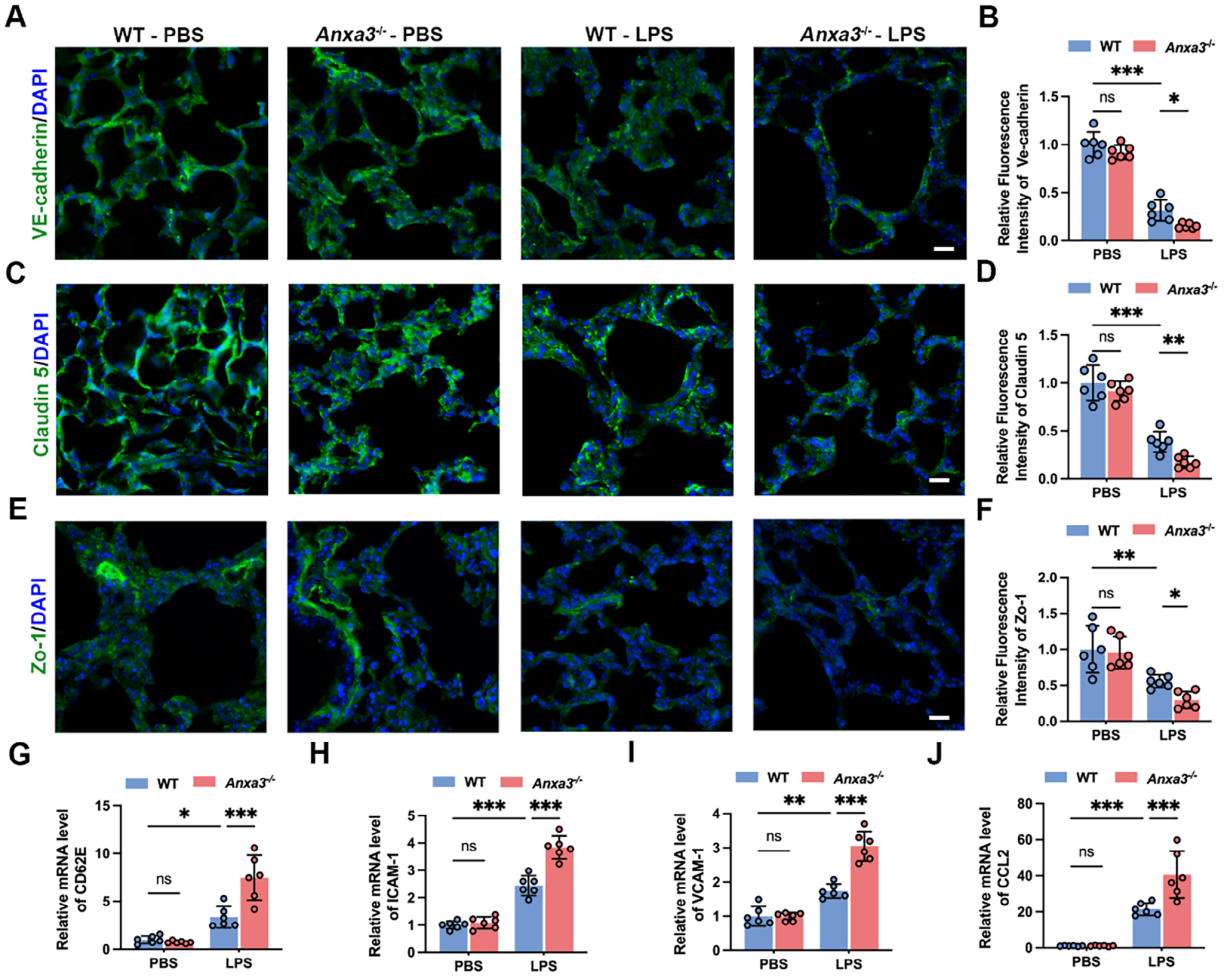
Knockout of ANXA3 exacerbates pulmonary vascular permeability and exacerbates inflammatory gene expression in LPS‐induced lung injury. IF staining analysis of the endothelial cell AJs protein VE‐cadherin A,B), the endothelial TJs marker claudin 5 C,D), and Zo‐1 E,F) in lung tissue sections from the indicated mice. G,J) mRNA levels of vascular activation markers (CD62E, ICAM‐1, VCAM‐1, and CCL2) in the lung tissues of the indicated mice. *n* = 6, ^*^ *p* < 0.05, ^**^ *p* < 0.01, ^***^ *p* < 0.001; two‐way ANOVA with Tukey's post hoc test.

We subsequently explored whether ANXA3 regulates the phenotype of endothelial cells in sepsis. Endothelial hyperpermeability is the main pathophysiological feature of ALI, and given that the vascular endothelial barrier is maintained by TJs and AJs, we assessed the endothelial AJs protein VE‐cadherin and the TJs proteins (Zo‐1 and claudin 5) by IF. The results showed that LPS exposure caused a reduction in VE‐cadherin (**Figure** **2**A,B), claudin 5 (Figure 2C,D) and Zo‐1 (Figure 2E,F) within the lung tissue, with these changes being more pronounced in ANXA3^−/−^ mice. These findings suggest that the loss of ANXA3 exacerbates the LPS‐induced disruption of the alveolar vascular barrier, resulting in increased vascular permeability and a more severe sepsis phenotype.

In sepsis, the activation of ECs into a phenotype, with the production of adhesion molecules, represents a key step in the development of sepsis‐induced endothelial dysfunction.^[^ ^35^ ^]^ We therefore examined the mRNA expression levels of adhesion molecules (CD62E, ICAM‐1, and VCAM‐1) and CCL2 in lung tissues by qRT–PCR. Our results revealed that ANXA3 deletion led to a significant increase in CD62E (Figure 2G), ICAM‐1 (Figure 2H), VCAM‐1 (Figure 2I), and CCL2 (Figure 2J) expression in lung tissues following LPS injection. These data suggest that ANXA3 can reduce endothelial activation and leucocyte adhesion, thus preventing the inflammatory cascade of sepsis‐induced lung injury.

### 2.3 ANXA3 Mediated‐EC Permeability is Mediated by Interactions with the Actin Cytoskeleton

**Figure 3:**
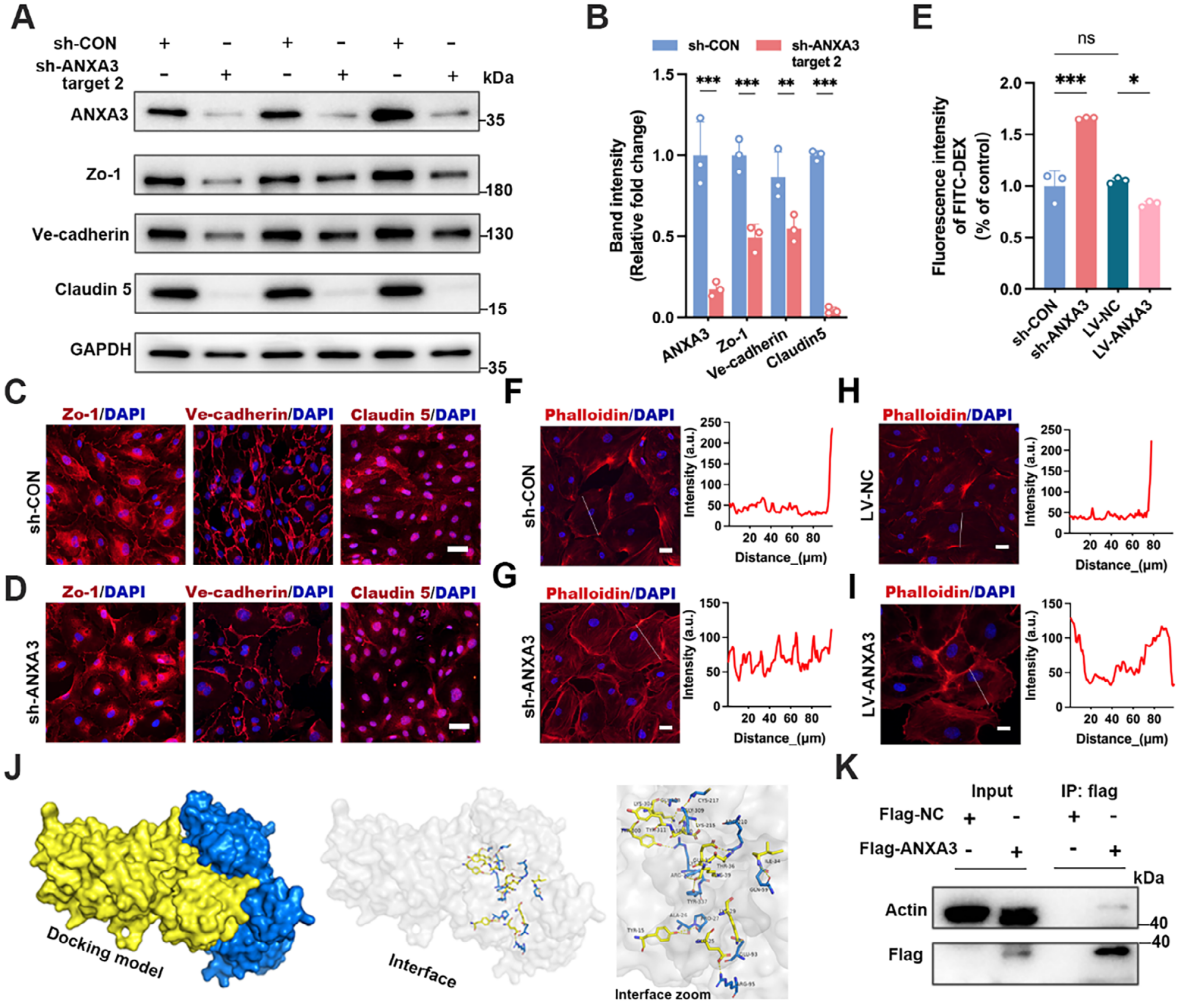
Role of ANXA3 in the regulation of junctional proteins. A,B) Gel images and quantification of WB showing ANXA3, Zo‐1, VE‐cadherin, and claudin 5 expression in HUVECs transiently infected with lentivirus containing a negative control sequence and a shRNA target‐2 sequence. *n* = 3, ^**^ *p* < 0.01, ^***^ *p* < 0.001, two‐tailed unpaired Student's *t* test. C,D) Immunocytochemical staining for ZO‐1, VE‐cadherin, and claudin 5 in HUVECs infected with lentivirus containing a negative control sequence C) or shRNA target‐2 sequence D). Scale bar = 50 µm, *n* = 4 independent experiments. E) After ANXA3 was knocked down or overexpressed in HUVECs, the fluorescence intensity of FITC‐DEX leakage from the upper chamber to the lower chamber in the transwell. *n* = 3, ^*^ *p* < 0.05, ^***^ *p* < 0.001. One‐way analysis of variance followed by Tukey's test was used for multiple group comparisons. F–I) Rhodamine‐phalloidin staining of the actin cytoskeleton in sh‐CON, sh‐ANXA3, LV–NC, and LV–ANXA3 cells (scale bar = 20 µm). Histograms represent intensity profiles of actin staining along the dotted lines in photomicrographs, emphasizing the differences in cross‐cellular actin expression. J) Molecular docking mimics the ANXA3 (yellow) and β‐actin (blue) complex. (K) Co‐IP experiments confirmed the interaction of ANXA3 with β‐actin.

To further explore how ANXA3 regulates endothelial function, we conducted in vitro experiments with primary HUVECs, which are powerful tools for studying ECs.^[^ ^36^ ^]^ We transiently infected HUVECs with lentivirus‐encapsulated short hairpin loop RNA (shRNA) to knock down ANXA3 expression (Figure S5A, Supporting Information). Compared with the scrambled shRNA control (sh‐CON), three shRNAs were found to induce varying extents of ANXA3 knockdown. Notably, the target‐2 shRNA had the highest knockdown efficiency, reaching 83%. This resulted in a reduction in Zo‐1 and VE‐cadherin expression of ≈50% and in claudin 5 expression of ≈95% in HUVECs (**Figure** **3**A,B). Target‐1 (Figure S5B,C, Supporting Information), and target‐3 shRNAs (Figure S5D,E, Supporting Information) caused an ≈50% reduction in ANXA3 expression, which led to less substantial decreases in the expression levels of Zo‐1, VE‐cadherin, and claudin 5 compared to the effects of target 2. These findings indicate that ANXA3 plays a pivotal and indispensable role in regulating the expression of Zo‐1, VE‐cadherin, and claudin 5. IF staining further revealed that ANXA3 knockdown disrupted the continuity of Zo‐1, VE‐cadherin, and claudin 5 at the cell membrane (Figure 3C,D). However, the overexpression of ANXA3 did not further increase the expression (Figure S6A,B, Supporting Information) of these proteins. We subsequently used a monolayer cell permeability assay to assess the influence of ANXA3 knockdown or overexpression on endothelial permeability comprehensively. Compared with that in the sh‐CON group, transient knockdown of ANXA3 led to a marked increase in transendothelial permeability, whereas ANXA3 overexpression decreased cell permeability (Figure 3E).

We further investigated how ANXA3 regulates endothelial intercellular junction proteins. The stability of tight and adherens junctions depends mainly on the actin cytoskeleton, especially its interaction with the cell membrane.^[^ ^7^ ^]^ Previous studies have suggested that ANXA3 colocalizes with actin in microglia.^[^ ^37^ ^]^ Immunostaining for F‐actin with rhodamine–phalloidin revealed that in static cultured control HUVECs, actin filaments are typically localized at the cell cortex, with occasional stress fibers observed on the apical side of the cell (Figure 3F,H). The knockdown of ANXA3 triggered substantial reorganization of F‐actin, leading to a notable increase in the formation of radial stress fibers (Figure 3G). Notably, as we used lentivirus‐packaged shRNA to transiently infect HUVECs, in some uninfected cells in which ANXA3 was not knocked down, there were no corresponding cytoplasmic radial stress fibers (Figure S7(indicated with arrows), Supporting Information). Conversely, the overexpression of ANXA3 markedly augmented cortical actin around the cell membrane, resulting in the formation of a well‐defined cortical actin ring. (Figure 3I). Given these significant alterations in the actin cytoskeleton, we sought to explore the relationship between ANXA3 and the actin cytoskeleton. Molecular docking predicted an interaction between ANXA3 and β‐actin (Figure 3J), and this was confirmed through biochemical co‐immunoprecipitation, which demonstrated a direct interaction between ANXA3 and β‐actin (Figure 3K).

### 2.4 ANXA3 Mediates Leukocyte Adhesion to ECs Through pATF2/CD62E Signaling

**Figure 4:**
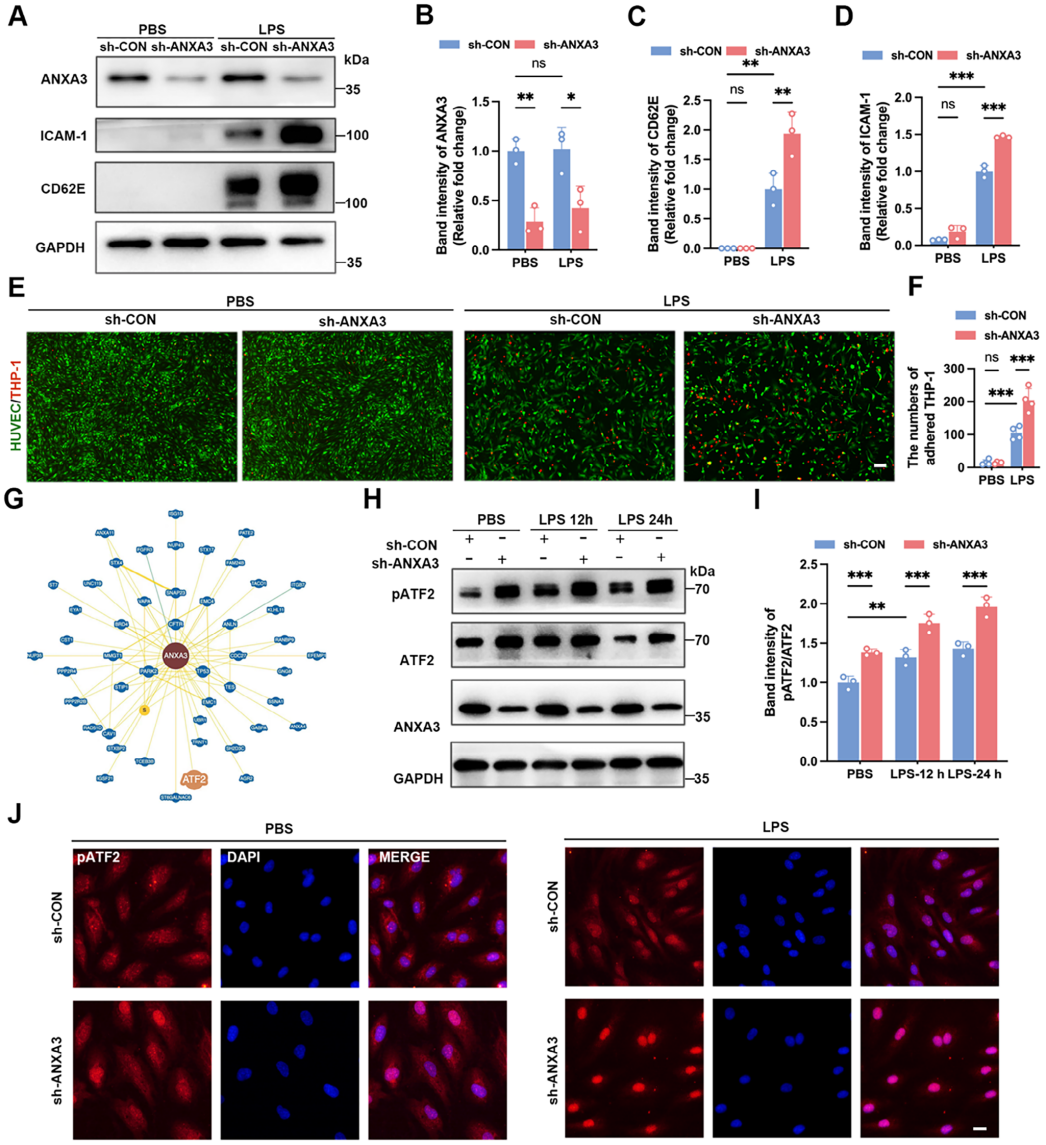
Knockdown of ANXA3 aggravates LPS‐induced endothelial activation. A–D) HUVECs were transiently infected with lentivirus containing a negative control sequence or shANXA3; at 48 h postinfection, the HUVECs were stimulated with LPS (1 µg mL^−1^) for 12 h. Immunoblots showing ICAM‐1 and CD62E expression after ANXA3 knockdown in HUVECs. *n* = 3, ^*^ *p* < 0.05, ^**^ *p* < 0.01, ^***^ *p* < 0.001; two‐way ANOVA followed by Tukey's test. E,F) Effects of ANXA3 knockdown on THP‐1 adhesion to HUVECs. Scale bar = 100 µm. G) The BioGRID (https://thebiogrid.org/) database was used to predict potential proteins that interact with ANXA3. H,I) Phosphorylation of ATF2 in control and ANXA3‐knockdown HUVECs was detected by WB. *n* = 3, ^**^ *p* < 0.01, ^***^ *p* < 0.001; two‐way ANOVA followed by Tukey's test.) pATF2 localization and fluorescence intensity were detected by IF in control and ANXA3‐knockdown HUVECs. Scale bar = 10 µm.

We revealed that ANXA3 knockout in mice led to increased expression of leukocyte adhesion molecules and leukocyte infiltration in the lungs in Sections 2.1 and 2.2. Therefore, we further investigated the role of ANXA3 in endothelial activation in vitro. Consistent with the in vivo findings, ANXA3 knockdown further increased the LPS‐induced upregulation of CD62E and ICAM‐1 (**Figure** **4**A–D) in HUVECs. These results were corroborated by IF detection of CD62E expression on the cell surface (Figure S8A, Supporting Information). Additionally, we observed that ANXA3 knockdown (Figure S8B, Supporting Information) heightened the inflammatory response to LPS stimulation, including increased transcription of the adhesion molecules ICAM‐1, VCAM‐1, and CD62E (Figure S8C–E, Supporting Information) and the chemokine CCL2 (Figure S8F, Supporting Information), and elevated mRNA levels of the proinflammatory cytokines TNF‐α and IL‐1β (Figure S8G–I, Supporting Information). Accordingly, the numbers of THP‐1 monocytic cells firmly adhering to HUVECs with ANXA3 knockdown was significantly greater than that in the control group (Figure 4E,F), suggesting that ANXA3 in ECs plays a role in inhibiting leukocyte adhesion during sepsis.

We next addressed the mechanism by which ANXA3 modulates LPS‐induced endothelial activation. The induction of inflammatory gene expression relies on the assembly of transcriptional complexes involving NF‐κB,^[^ ^38^ ^]^ AP‐1,^[^ ^10^ ^]^ and others. Among these complexes, the activation of the TLR4/NF‐κB signaling pathway by LPS is the most extensively characterized pathway;^[^ ^39^ ^]^ therefore, we initially investigated whether ANXA3 knockdown influences NF‐κB activation. Our results revealed that LPS increased the level of phosphorylated P65, however, ANXA3 knockdown did not affect P65 phosphorylation (Figure S8J,K), suggesting that ANXA3 does not influence NF‐κB activation. ATF2, an important member of the AP‐1 complexes, serves as a key regulator in controlling the expression of CD62E.^[^ ^40^ ^]^ The protein interaction database BioGRID predicted that ANXA3 may interact with ATF2 (Figure 4G). Phosphorylation of ATF2 (pATF2) at threonine 71 is necessary for its activation and subsequent binding to the PDII site in the CD62E promoter.^[^ ^41^ ^]^ We found that ANXA3 knockdown increased pATF2 and further intensified LPS‐induced pATF2 (Figure 4H,I). IF staining also revealed that ANXA3 knockdown alone not only increased the fluorescence intensity of pATF2 but also promoted its nuclear translocation. After LPS stimulation, pATF2 was detected within the nucleus, with more pronounced fluorescence intensity in ANXA3 knockdown HUVECs (Figure 4J).

**Figure 5:**
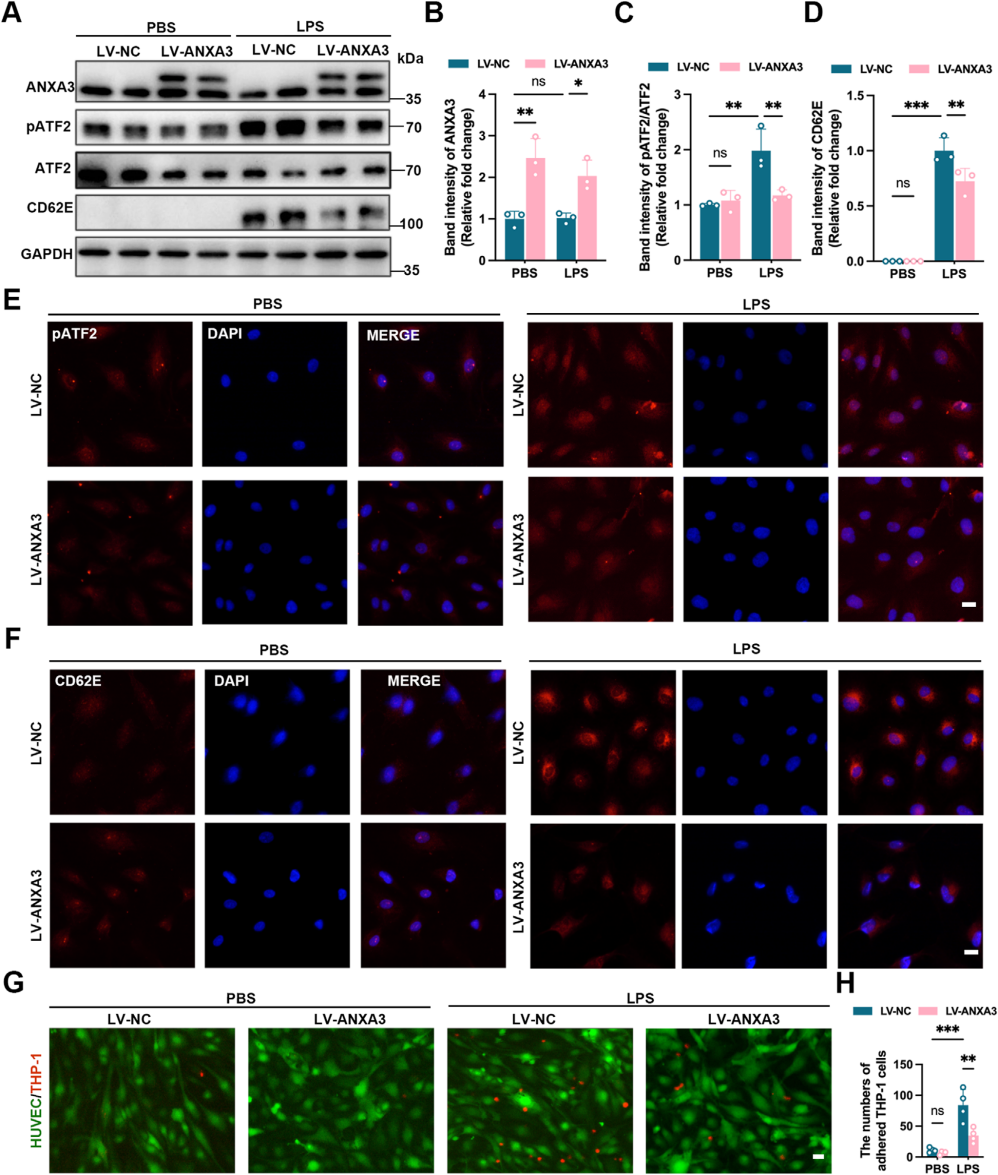
Overexpressed ANXA3 limits LPS‐induced endothelial activation. A–D) Control and LV–ANXA3 HUVECs were stimulated with LPS and immunoblotting revealed pATF2 and CD62E expression. *n* = 3, ^*^ *p* < 0.05, ^**^ *p* < 0.01, ^***^ *p* < 0.001; two‐way ANOVA followed by Tukey's test. E,F) The localization and fluorescence intensity of pATF2 and CD62E in LV–NC and LV–ANXA3 HUVECs before and after LPS stimulation were detected by IF staining. Scale bar = 10 µm. G,H) Effects of ANXA3 overexpression on THP‐1 adhesion to HUVECs. Scale bar = 20 µm. ^***^ *p* < 0.001, two‐way ANOVA followed by Tukey's test. [Correction added on a 8 April 2025, after first online publication: Figure 5 image is replaced with the current version.]

Next, we examined the effect of ANXA3 overexpression on LPS‐induced EC activation. The results revealed that ANXA3 overexpression (**Figure** **5**A,B) led to an ≈60% reduction in pATF2 (Figure 5A,C) and a significant decrease in CD62E expression in HUVECs (Figure 5A,D). IF staining analysis further revealed that ANXA3 overexpression partially reduced the fluorescence intensity of LPS‐induced pATF2 phosphorylation (Figure 5E). In addition, ANXA3 overexpression inhibited LPS‐induced CD62E expression on the surface of HUVECs (Figure 5F) and decreased the numbers of LPS‐induced THP‐1 monocytes adhering to HUVECs (Figure 5G,H).

### 2.5 Inhibition of pATF2 Suppresses the Endothelial Activation Caused by ANXA3 Knockdown and Prevents the Death of Septic Mice Resulting from ANXA3 Knockout

**Figure 6:**
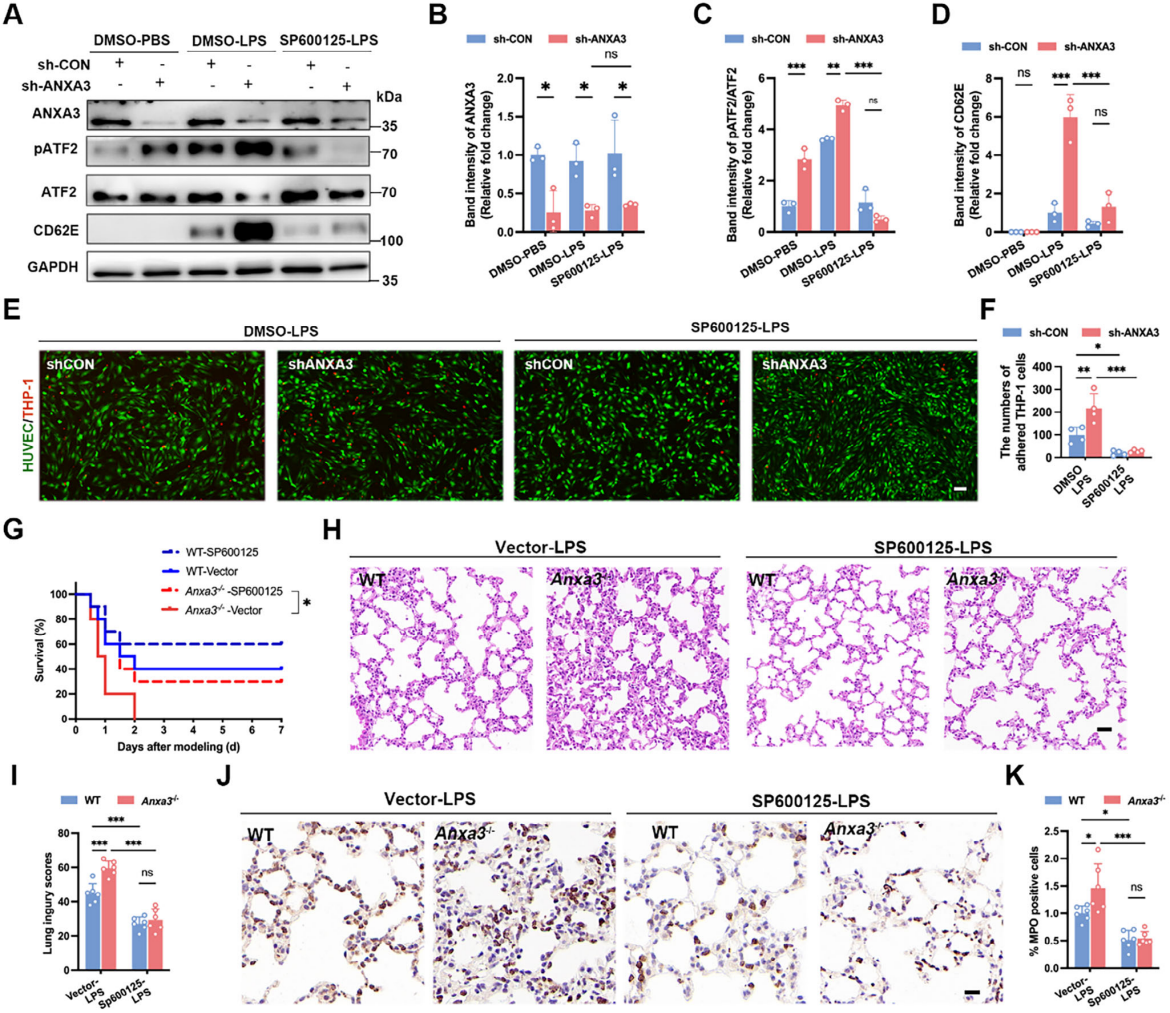
The pATF2 inhibitor SP600125 rescues ANXA3 loss‐mediated in vitro endothelial activation and in vivo lung injury during sepsis. A–D) shCON‐ and shANXA3‐treated HUVECs were pretreated with SP600125 for 1 h and then stimulated with LPS, after which the protein levels of pATF2 and CD62E were detected by WB. *n* = 3, ^*^ *p* < 0.05, ^**^ *p* < 0.01, ^***^ *p* < 0.001; two‐way ANOVA followed by Tukey's test. E,F) shCON‐ and shANXA3‐treated HUVECs were pretreated with SP600125, and THP‐1 adhesion to HUVECs was observed after LPS stimulation. Scale bar = 100 µm, *n* = 4; ^***^ *p* < 0.001; two‐way ANOVA followed by Tukey's test. G) The SP600125 inhibitor was used 1 h before and 12 h after LPS modeling, and survival analysis was performed after LPS treatment. Comparisons of survival curves between untreated and SP600125‐treated ANXA3^−/−^ mice (^*^ *p* < 0.05) via the log‐rank (Mantel–Cox) test. H,I) H&E staining and lung injury scores of lung tissue from ANXA3^−/−^ and WT male mice treated with SP600125 and vector 18 h after LPS modelling. Scale bar = 20 µm. *n* = 6, ^***^ *p* < 0.001; two‐way ANOVA followed by Tukey's test. J,K) MPO staining and quantitative analysis of lung tissues from ANXA3^−/−^ and WT male mice treated with SP600125 and vector 18 h after LPS modelling. Scale bar = 10 µm. *n* = 6, ***p < 0.001, ^*^ *p* < 0.05; two‐way ANOVA followed by Tukey's test.

To further demonstrate the involvement of ATF2, we used the pharmacological inhibitor SP600125 to suppress pATF2 n in shANXA3‐induced EC activation. The results revealed that ANXA3 knockdown led to elevated CD62E expression in HUVECs under LPS stimulation, whereas pretreatment with SP600125 effectively inhibited the increase in CD62E expression (**Figure** **6**A–D). Moreover, SP600125 pretreatment also decreased the increased adhesion of THP‐1 monocytes induced by ANXA3 knockdown (Figure 6E,F).

To address whether ATF2 is required and sufficient for the LPS‐induced increase in lung inflammation in ANXA3‐knockout mice, WT, and ANXA3^−/−^ mice were pretreated with SP600125, which partially improved the survival rate of ANXA3^−/−^ mice from 0 to 30% (Figure 6G). Additionally, SP600125 alleviated the LPS‐induced lung injury caused by ANXA3 deletion (Figure 6H,I) and reduced leukocyte infiltration in the lung tissues of ANXA3 knockout mice (Figure 6J,K).

### 2.6 Destabilization of Actin Filaments Selectively Inhibits shANXA3‐induced ATF2 Activity and CD62E Expression in Endothelial Cells

Previous studies have shown that the cytoskeleton, as an important modulator of intracellular signaling, is involved in the signaling of pATF2 in ECs.^[^ ^42^ ^]^ Moreover, other reports have indicated that the proinflammatory phenotypic differentiation of ECs is correlated with stress fiber polymerization of the cytoskeleton.^[^ ^12^, ^43^ ^]^ In our research, we have observed that ANXA3 can influence the reorganization of the cellular actin cytoskeleton. On the basis of these previous findings and our observations, we hypothesized that the actin cytoskeleton might play a crucial role in the increased activation of ATF2 in endothelial cells during sepsis induced by ANXA3 knockdown.

**Figure 7:**
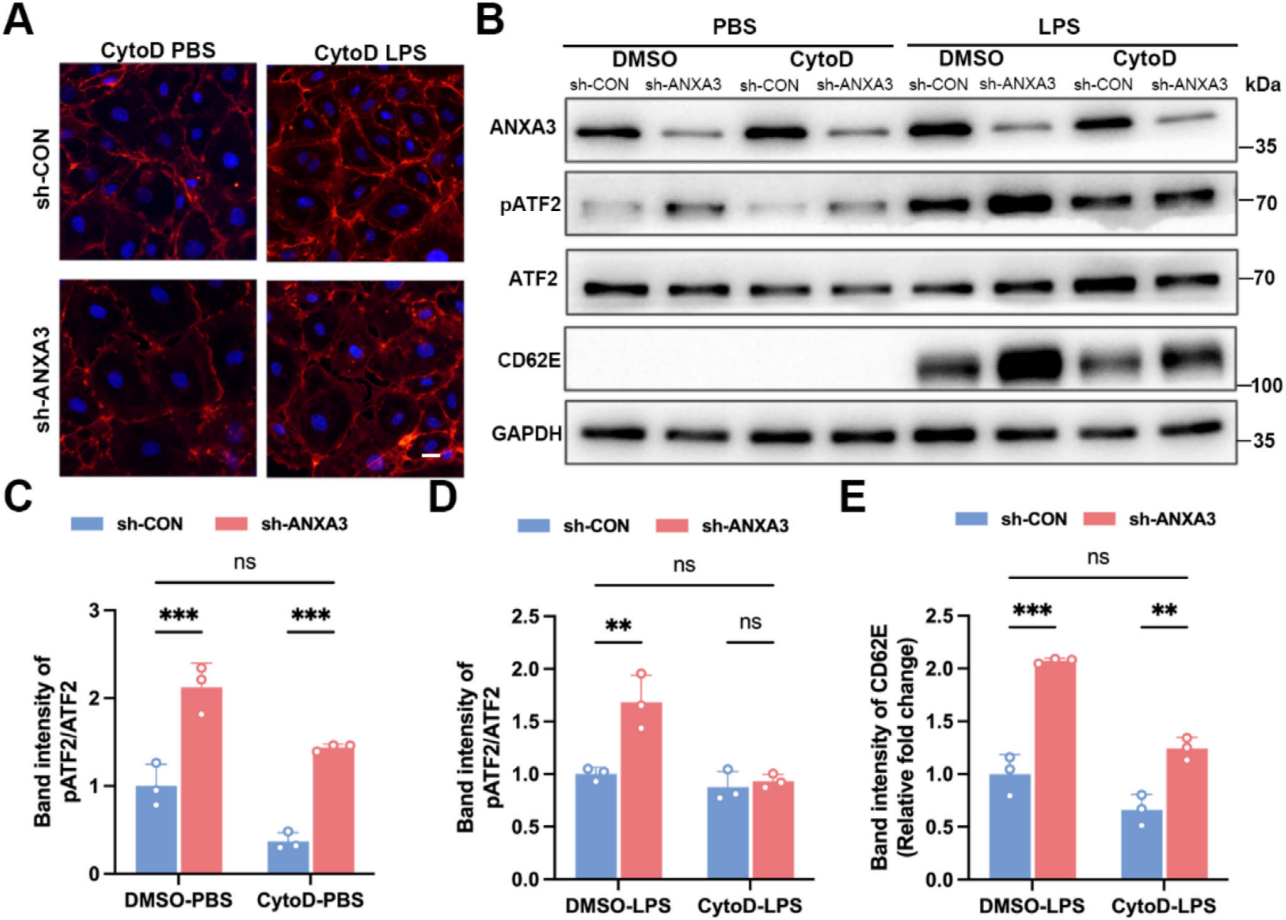
Cyto D inhibits the increased level of pATF2 induced by ANXA3 knockdown. A) HUVECs in the sh‐CON and sh‐ANXA3 groups were pretreated with Cyto D and then treated with LPS or PBS respectively. The actin cytoskeletons were detected by phalloidin staining. Scale bar = 10 µm. B) Gel images showed the effect of Cyto D on pATF2/CD62E levels in ANXA3‐knockdown cells and control cells, and on pATF2/CD62E levels in both groups after LPS stimulation. C) Statistical analysis of the pATF2 in ANXA3‐knockdown cells and control cells treated with Cyto D. *n* = 3, ^***^ *p* < 0.001, two‐way ANOVA followed by Tukey's test was used for multigroup comparison. D,E) Statistical analysis of the pATF2/CD62E levels in ANXA3 knockdown cells and control cells pretreated with Cyto D then stimulated with LPS. *n* = 3, ^**^ *p* < 0.01, ^***^ *p* < 0.001, two‐way ANOVA followed by Tukey's test was used for multigroup comparison.

Pretreatment with cytochalasin D (Cyto D) prevented the formation of stress fibers induced by ANXA3 knockdown and inhibited LPS stimulation‐induced cytoskeletal stress fibers (**Figure** **7**A). Cyto D also inhibited ANXA3 knockdown‐induced pATF2 activation in ECs and partially inhibited LPS‐induced pATF2 activation (Figure 7B,C) and CD62E expression (Figure 7B,D). These findings suggest that LPS activates pATF2 through both actin‐dependent and actin‐independent pathways. Importantly, Cyto D suppressed the increase in pATF2 activation (Figure 7B,D) and CD62E expression (Figure 7B,E) in ANXA3‐knockdown HUVECs following LPS stimulation.

## 3 Discussion

**Figure 8:**
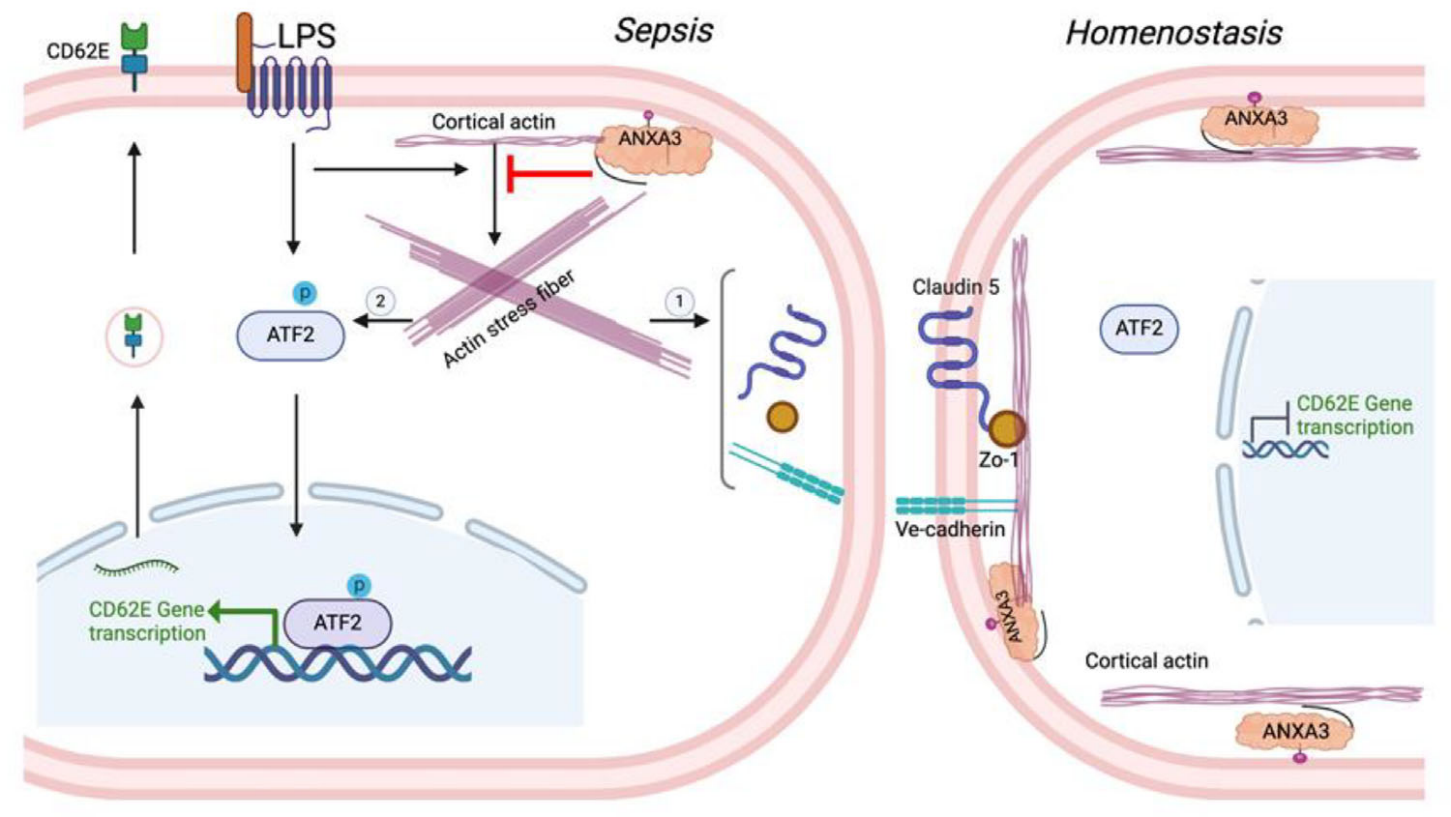
Schematic diagram of ANXA3 plays a protective role in endothelial function in sepsis.

In this study, we conducted in vitro and in vivo experiments for an in‐depth exploration of the effects of ANXA3 on EC function during sepsis and its underlying mechanisms. In vitro experiments demonstrated that ANXA3 knockdown in primary HUVECs triggered a significant increase in intracellular actin stress fibers, resulting in a reduction in TJs and AJs proteins and a subsequent increase in cell permeability. Additionally, ANXA3 knockdown increased the LPS‐induced phosphorylation of ATF2, which in turn promoted the overexpression of CD62E and led to an increase in leukocyte adhesion (**Figure** **8**). In vivo, compared with WT mice, ANXA3 knockout mice exhibited increased vascular permeability and leukocyte infiltration in a sepsis model, leading to more severe lung injury and ultimately to increased mortality.

Members of the annexin family are extensively expressed within the cardiovascular system of vertebrates, particularly in mammals. Conversely, in the embryos of Xenopus and zebrafish, only one or a few annexins^[^ ^30^ ^]^ are expressed in ECs. Notably, ANXA3 appears to be the only member expressed in both higher vertebrates (such as humans and mice) and lower vertebrates (such as Xenopus and zebrafish),^[^ ^30^ ^]^ indicating that ANXA3 might play a pivotal, evolutionarily relevant role in vascular biology. In this study, we found that ANXA3 knockout did not influence the expression of VE‐cadherin, the tight junction protein Zo‐1, or claudin 5 in mice, however, when ANXA3 was transiently knocked down in HUVECs, the expression levels of VE‐cadherin, Zo‐1, and claudin 5 subsequently decreased. This may be because the formation of the vascular endothelial barrier in mice is related to multiple cellular and protein interactions, and ANXA3 knockout may result in adaptive changes that do not affect endothelial cell connectivity and permeability under normal conditions. However, upon LPS stimulation, damage to AJs and TJs proteins increased, and permeability also increased. Other family members, such as ANXA2, behave similarly, the blood–brain barrier in ANXA2 knockout mice develops slowly but can still form eventually.^[^ ^22^ ^]^ However, in the case of traumatic brain injury, ANXA2 knockout mice exhibit more severe damage because the knockout increases the vulnerability of the BBB.^[^ ^44^ ^]^

Leukocyte adhesion is a key component in the onset and progression of sepsis.^[^ ^45^ ^]^ To investigate the effects of ANXA3 on local vascular inflammation, we studied the expression of adhesion molecules and their corresponding transcription factors. The results showed that ANXA3 knockdown significantly promoted CD62E expression, which was pATF2‐associated rather than NF‐κB‐associated. CD62E, one of the very few proteins that are highly restricted to ECs,^[^ ^46^ ^]^ causes leukocytes to roll slowly on the EC surface as the first step in adhesion. ATF2 is the specific transcription factor for CD62E.^[^ ^47^ ^]^ The overexpression of ANXA3 in HUVECs inhibited ATF2 phosphorylation, thus suppressing CD62E expression and reducing LPS‐induced endothelial activation. Collectively, these findings suggest that ANXA3 has potential value in the treatment of sepsis and we are currently studying on this topic.

The actin cytoskeleton is crucial for the structure and function of ECs and performs essential mechanical, organizational, and signaling functions.^[^ ^48^ ^]^ It is a highly dynamic structure and undergoes polymerization and depolymerization based on cellular demand. It organizes into three distinct structures, including the membrane skeleton, the cortical actin rim, and stress fibers. Actin dynamics are essential for regulating endothelial barrier function^[^ ^49^ ^]^ and EC activation;^[^ ^11^, ^12^ ^]^ endothelial integrity requires a balance between the formation of cortical actin filaments that maintain endothelial cell contact stability and the formation of actin stress fibers that generate pulling forces and thus increase cell permeability.^[^ ^49^ ^]^ We found that the overexpression of ANXA3 caused more pronounced staining of cellular cortical actin, suggesting that ANXA3 promotes cortical actin assembly and thereby increases the endothelial barrier. In contrast, ANXA3 knockdown resulted in the development of stress fibers that compromised endothelial cell contact stability and increased intercellular permeability. Furthermore, the use of Cyto D to inhibit the formation of stress fibers in shANXA3‐knockdown ECs suppressed the increased phosphorylation of ATF2 and the increased expression of CD62E induced by LPS stimulation.

Actin interacts with many secondary proteins and its dynamics are highly controlled by the nature of these interactions. Annexins have been shown to interact directly with actin.^[^ ^14^, ^15^ ^]^ ANXA2 was originally identified as an F‐actin‐interacting protein,^[^ ^50^ ^]^ it is recruited to the site of actin assembly at cell membranes by binding G‐ and F‐actin to affect cytoskeletal remodeling.^[^ ^51^ ^]^ In cells lacking ANXA2, F‐actin is disorganized.^[^ ^52^ ^]^ Subsequent studies have demonstrated that other ANXAs including ANXA1, ANXA5, ANXA6, and ANXA8^[^ ^53^ ^]^ can also bind and bundle actin filaments in a Ca^2+^‐dependent manner and play essential roles in membrane dynamics.^[^ ^14^, ^54^ ^]^ Previous studies have also shown that in microglia, ATP treatment induces ANXA3 translocation to ruffled membranes with calcium‐dependent F‐actin‐binding activity.^[^ ^37^ ^]^ Our study indeed revealed that ANXA3 interacted with actin in ECs and that the knockdown and overexpression of ANXA3 significantly affected the actin cytoskeleton. We also found that ANXA3 influenced the activation of ATF2, and previous studies have shown that the prominent role of ATF2 in endothelial proinflammatory gene expression is associated with the structure of the actin cytoskeleton.^[^ ^42^, ^55^ ^]^ In the current study, the use of Cyto D to block the actin polymerization induced by ANXA3 knockdown led to a marked inhibition of LPS‐induced ATF2 phosphorylation and CD62E expression. Collectively, these findings clearly establish a prominent role for ANXA3 in modulating many unexpected aspects of EC dysfunction through the regulation of the actin cytoskeleton.

The study revealed that ANXA3 deficiency resulted in increased mortality in male mice but not in female mice. Sex differences have been a significant concern in sepsis research.^[^ ^56^ ^]^ Epidemiological studies have shown that females tend to be more resistant to infection and sepsis than males are, although they are more susceptible to autoimmune diseases.^[^ ^57^ ^]^ In the CLP‐induced polymicrobial sepsis model, premetamorphic female mice presented significantly higher survival rates than did male mice,^[^ ^58^ ^]^ and injury severity was markedly greater in male and ovary‐denuded mice than in ovary‐intact female mice.^[^ ^59^ ^]^ Sex differences have also been reported in studies on the annexin family. ANXA1, a well‐known anti‐inflammatory protein, has been shown to interact with estrogens, potentially contributing to sexual dimorphism in systemic inflammatory responses.^[^ ^60^ ^]^ These findings provide a promising direction for future research.

Our study revealed no significant difference in survival between the WT and ANXA3^−/−^ groups when 15 mg kg^−1^ LPS was used for sepsis modeling. However, the ANXA3^−/−^ mice presented a more pronounced decrease in body temperature at 12 h postmodeling. Body temperature is a critical indicator in mouse models of sepsis, as septic mice typically develop hypothermia when kept at room temperature,^[^ ^61^ ^]^ and the severity of the temperature decrease is correlated with disease severity.^[^ ^62^ ^]^ Our findings suggest that ANXA3^−/−^ mice exhibited a greater response to sepsis than did WT mice in the early stages. The 15 mg kg^−1^ LPS dose represents a substantial challenge, and assessing survival only at long‐term intervals may have masked early differences between the groups. This study highlights, through the use of different sepsis models and LPS doses, that ANXA3 deficiency amplifies the response of mice to sepsis. Furthermore, the observed phenotypic differences between male and female mice could provide a promising direction for future research.

Although this study has improved our understanding of the role of ANXA3 in maintaining EC permeability and inflammation, it had some limitations. First, owing to resource limitations, we used only global gene knockout mice in our in vivo experiments and did not used endothelial cell‐specific knockout mice. We supplemented the results with studies using endothelial cells in vitro. Second, in rescue experiments, the use of a stabilizer of the actin cytoskeleton is preferable. However, we did not find the appropriate drugs. However, the functions of the recombinant ANXA3 protein and mimetic peptide as actin stabilizers should be further investigated. Finally, this study used primary HUVECs for in vitro experiments, which make the research results more relevant to clinical practice and have positive implications for clinical translation. However, there are still some differences between primary HUVECs and organ‐specific microvascular endothelial cells, which may affect the extrapolation of the research results to some extent.

In summary, ANXA3 plays an endogenous protective role in endothelial dysfunction in sepsis by modulating endothelial cell permeability and inhibiting pATF2/CD62E inflammatory signaling through stabilizing the actin cytoskeleton (Figure 8).

ANXA3 plays an endogenous protective role in endothelial dysfunction in sepsis by stabilizing the actin cytoskeleton, modulating cell permeability, and inhibiting pATF2/CD62E inflammatory signaling. The graphics of Figure 8 and ToC were created and licensed with BioRender.com.

## 4 Experimental Section

### Mice

ANXA3 knockout mice were purchased from Cyagen Biosciences (Suzhou, China). To create ANXA3 knockout mice, gRNA‐directed Cas9 endonuclease cleaved the ANXA3 gene at exons 3 and 6 and created double‐strand breaks. These breaks resulted in the disruption of ANXA3. All the mice in this study were bred on a C57BL/6N background, and both females and males were included. Ethical permits (2021sydw0027) were approved by the Ethics Committee of Central South University and all experiments were carried out in accordance with the guidelines of the National Institutes of Health of China Guide for the Care and Use of Laboratory Animals.

### Genotyping

All mice were genotyped prior to use. Cut ≈2 mm of mouse tail tissue and placed it into a 1.5 ml centrifuge tube. Added 80 µL of 50 mm NaOH solution (Sinopharm chemical reagent, China) and incubated it in 100 °C for 30 min. Added 40 µL of Tris–HCl buffer (pH 7.5) (Biosharp, China). Subsequently, centrifuged and collected the supernatant, which represented the genomic DNA. Thereafter, utilized the genomic DNA for PCR amplification. The amplification reaction system consisted of 1.25 µL of genomic DNA, 10 µL of 2 × Taq Master Mix (Vazyme, China), 7.15 µL of ddH_2_O, 0.8 µL of forward primer, and 0.8 µL of reverse primer (10 µm L^−1^). The PCR cycling conditions were as follows: initiated with one cycle at 94 °C for 3 min; then performed 35 cycles, each cycle involved 30 sec at 94 °C, 35 sec at 60 °C, and 35 sec at 72 °C; finally, conducted an extension step at 72 °C for 5 min. Under these conditions, the WT allele yielded a 641 bp band, while the homozygous knockout allele generated a 585 bp band, the heterozygous generated both the 641 and the 585 bp bands. The following PCR primer sequences were used for analysis: forward primer as 5′‐AAGCTCCAAGATGTCAGCAGAG‐3′, reverse primer 1 as 5′‐ACTTAGTCCTTGGTTCTTGGGAC‐3′, reverse primer 2 as 5′‐ATCCCTGAGGCTCTGAATTTGC‐3′. The primers were synthesized by Sangon Biotech.

### Mouse Models of Endotoxemia and Polymicrobial Sepsis

All the mice used in the experiments were littermates and age‐matched ANXA3^−/−^ and WT mice. Endotoxemia was induced by intraperitoneal injection of LPS (10 or 15 mg kg^−1^). The clinical behavior score^[^ ^63^ ^]^ was used to monitor the condition of the mice. The scoring criteria included the following items: the appearance, activity, consciousness, eye condition of the mice, as well as the respiratory rate and quality. The maximum score for each item was 4 pts. The core temperature was measured with a rectal thermoprobe (Deli, China).

A sublethal model of CLP was used according to a description published previously.^[^ ^17^ ^]^ Briefly, the mice were anesthetized with 1% sodium pentobarbital (50 mg kg^−1^) by intraperitoneal injection, and the abdomen was shaved and disinfected. The cecum was then identified and exposed, ligated in the outer third of the cecum and punctured with a 21‐gauge needle. The abdominal muscles and skin were then closed with simple sutures. In sham mice, only the cecum was exposed but not punctured, and then the cecum was placed back into the abdominal cavity.

### Histological Analysis

The mice were anesthetized and their trachea was ligated. Then, the mice were perfused free of blood with cold PBS, and the lung, kidney, liver, spleen, and intestine tissues were fixed with 4% polyformaldehyde (PFA). PFA‐fixed tissues were washed with PBS and dehydrated in gradient ethanol followed by paraffin processing. The lungs were cut into 4–5 µm thick sections and stained with H&E. Pictures were taken with a digital slide scanner CaseViewer (Budapest, Hungary).

### Lung Injury Score

Ten fields of view were randomly selected, after which the fields of view were scored blindly according to the criteria recommended by the official workshop of the American Thoracic Society^[^ ^64^ ^]^ on the basis of the following five parameters on a scale of 0–2: A) intra‐alveolar neutrophils, B) interstitial neutrophils, C) hyaline membranes, D) exudation of protein fragments from the airway, and E) thickening of the alveolar septa.

### Measurement of Vascular Leakage of the Lung–Evans Blue (Eb) Dye Leakage

The tail veins of the mice were injected with 1% EB (4 mL kg^−1^) 1 h before lung collection.^[^ ^65^ ^]^ The tissues were perfused with PBS, after which the lungs were dried at 60 °C for 72 h to obtain the dry weight. 500 µL of formamide (Macklin, China) were added, and the mixture was incubated at 60 °C for 24 h. The absorption of the supernatants was determined at 620 nm. The concentration of EB dye was calculated with a standard curve. The Evans blue index was calculated as the amount of EB dye in formamide relative to the dry weight of the lung tissue.

### FITC‐Dextran Leakage

The mice were anesthetized and injected with 100 µL of 2 mg mL^−1^ 70 kDa FITC‐dextran (Sigma–Aldrich, USA).^[^ ^66^ ^]^ 1 h after the injection, the mice were perfused with PBS. The left lung was harvested, fixed in paraformaldehyde, and embedded in Tissue‐Tek O.C.T. compound (Sakura Finetek, Japan). The lung tissue was then cryosectioned and stained with DAPI (SouthernBiotech, USA). FITC‐dextran leakage within the lung tissue was observed by laser microscopy.

### Cell Culture

Primary HUVECs (DFSC–EC–01) were purchased from Cell Research (Shanghai, China) and were cultured in endothelial cell medium (Cell Research, China) supplemented with 5% FBS, 1% endothelial cell growth supplement, and 1% penicillin/streptomycin. HUVECs were used between passages 3 and 8. THP‐1 cells (TCH‐C361) were purchased from Hysigen Bioscience (Suzhou, China) and were cultured in RPMI‐1640 with 10% FBS, 0.05 mm β‐mercaptoethanol and 1% penicillin/streptomycin (Hysigen Bioscience, China).

### Lentiviral Infection of HUVECs

Up‐ and downregulation of ANXA3 expression in HUVECs were achieved via lentiviral infection (LV–ANXA3 and LV–shANXA3, respectively) as previously described.^[^ ^24^ ^]^ ANXA3 overexpression was performed by infecting HUVECs with the lentiviral vector GV492 expressing ANXA3 cDNA (LV–ANXA3), and the control group was LV–NC. For ANXA3 knockdown, three different shRNAs (sh‐ANXA3 targets 1–3) were used, targeting human ANXA3 (Gene Bank Accession: NM_0 05139), which were cloned and inserted into GV493 (sequences were shown in Table S1, Supporting Information). To better investigate the role of ANXA3 in cells, lentiviral particles with shRNAs or a scrambled sequence (sh‐NC; 5′‐TTCTCCGAACGTGTCACGT‐3′) as a control were used to infect HUVECs transiently. Briefly, the cells were grouped and seeded in dishes, and after 24 h, the lentivirus was added to infect the cells (multiplicity of infection (MOI) = 10). The culture medium was replaced 24 h after viral infection, and 48 h after medium replacement, the cell modeling treatment was performed. All the lentiviral particles were synthesized by Genechem (Shanghai, China). Up‐ and downregulation of ANXA3 protein expression were identified by WB.

### Cell Treatment

LPS (InvivoGen, USA) was reconstituted in PBS and used at a final concentration of 1 µg mL^−1^ in the culture medium during static culture. Sp600125 (MCE, China) was reconstituted in DMSO (MCE, China) and used at a final concentration of 50 nm L^−1^ to treat cells for 1 h. CytoD (MCE, China) was reconstituted in DMSO, and used at a final concertation of 2 nm L^−1^ to treat the cells for 15 min.

### RNA Isolation, Reverse Transcription, and Quantitative Real‐Time PCR

Total RNA was extracted from lung tissues and HUVECs with the Transzol‐up reagent (TransGen Biotech, China), and cDNA was generated from 1 µg of total RNA with a Transcriptor First Strand cDNA Synthesis Kit (TransGen Biotech). The expression level of RNAs was quantified with the FastStart Universal SYBR Green Master Kit (TransGen Biotech) on a real‐time system (ABI, USA). The relative transcript levels of three technical repeats per sample were normalized to the geometric mean of amplification of GAPDH, with expression levels calculated by the 2–ΔΔCT method. All primers were synthesized by Sangon Biotech (Shanghai, China). The sequences of the primers used for qRT–PCR were presented in Tables S2 and S3.

### Western Blotting (WB)

For the protein analysis of mouse organ tissues, after the animals are euthanized, 10–20 mL of ice‐cold PBS was perfused throughout the body, and then their organs were rapidly frozen. Tissue lysis was performed by homogenizing 100 mg of snap‐frozen tissue in 150 µL of RIPA buffer (NCM, China) containing a 1% phosphatase inhibitor and protease inhibitor cocktail (NCM, China). The lysates were centrifuged at 13000 × g for 15 min, the supernatants were collected, and the concentration was quantitated by the BCA method (NCM, China). The samples were boiled for 10 min, and equal amounts were applied to 4–20% SDS–polyacrylamide gels (Beyotime Biotechnology, China), electrophoresed and transferred to a PVDF membrane (Millipore, Germany). The membrane was blocked in 5% (w/v) milk in TBST for 1 h, followed by incubation with primary antibodies at 4 °C overnight and with horseradish peroxidase (HRP)‐conjugated secondary antibodies for 1 h at room temperature (RT). After being washed three times with TBST, the samples were visualized with enhanced chemiluminescence (ECL) reagent (Beyotime Biotechnology, China) with an ImageQuant 800 (Cytiva, USA). For two target molecules with close molecular weights, usually, after the bands had been exposed, used the membrane stripping solution (Abiowell, China) to strip the antibodies on the membrane for 15 min. After that, blocked the bands, continued to incubate with a new primary antibody, and the steps of incubating with the secondary antibody and exposing were repeated. Where indicated, the band intensity was quantified by densitometry with Fiji/ImageJ. The band intensity of the protein of interest was normalized to that of the corresponding loading control (GAPDH).

For protein analysis, the cells were washed in ice‐cold PBS and lysed on ice in RIPA buffer supplemented with protease inhibitors and a phosphatase inhibitor cocktail. For the detection of membrane proteins, protein samples were denatured at room temperature for 15 min. The remaining steps were as described above.

### Immunofluorescence Staining

For immunofluorescence staining, HUVECs were grown on recombinant fibronectin (rFN) (Cell Research, China)‐coated glass coverslips. For the staining of membrane proteins, cells were fixed in 4% PFA at 37 °C for 15 min. For the staining of pATF2, cells were fixed in pre‐cooled methanol at −20 °C for 5 min. The cells were incubated with primary antibody overnight at 4 °C. Alexa 594‐labeled secondary antibodies were used for 1 h at room temperature, followed by DAPI nuclear staining, mounting, and fluorescence microscopy (Nikon, Japan) analysis. Images were processed with Adobe Photoshop software.

### Phalloidin Staining

HUVECs were grown on rFN‐coated glass coverslips and fixed with 4% PFA. Then, 100 µL of 1× Texas Red Phalloidin (Abcam, England) solution was added to the samples, which were incubated for 1 h at RT. The slides were then washed with PBS for three times, and 15–20 µL of DAPI‐containing sealer was added to the slides, which were then observed under a fluorescence microscope.

### Cellular Permeability Assay

HUVECs were grown to confluence in 24‐well transwell chambers precoated with rFN. ANXA3 knockdown and overexpression were performed as described above. 100 µL of Hanks balanced salt solution (HBSS) containing 1 mg mL^−1^ 40 kDa FITC‐dextran (Thermo, USA) was added to the upper chamber, and 600 µL of HBSS was added to the lower chamber. After incubation for 1 h in an incubator protected from light, the fluorescence intensity of each lower chamber was detected by setting the excitation light at 493 nm and the emission light at 518 nm in a fluorescence enzyme (PerkinElmer, England) to react with the permeation by the fluorescence intensity.

### Molecular Docking

The docking models of the ANXA1–ACTB complex were generated with the Phyre2 web server for protein–protein docking (http://www.sbg.bio.ic.ac.uk/phyre2/html/page.cgi?id=index) based on the crystal structures of ACTB (Protein Data Bank code P60709) and ANXA3 (P12429). Interacting residues were visualized on the Protein Data Bank in Europe (https://www.ebi.ac.uk/pdbe/pisa/). Structural illustrations were prepared with PyMOL Molecular Graphic Systems (version 2.5.4, Schrödinger LLC; http://www.pymol.org/).

### Immunoprecipitation

The LV—Flag–NC and LV—Flag–ANXA3 cells were harvested with NP–40 lysis buffer (Abiowell, China) and then incubated with magnetic beads labeled with anti‐Flag antibody (NuoyiBio, China) at 4 °C for 2 h. 20 µL of 2x sampling buffer were added to the magnetic beads, which were then heated at 100 °C for 10 min to elute the protein in the magnetic beads. WB was used to assess the immunoprecipitated protein.

### THP‐1 Adhesion Assay

Each group of HUVECs was cultured in 12‐well plates and treated according to the conditions of each group. THP‐1 cells were labeled with 5 µg mL^−1^ Dil (Keygen, China) for 30 min and then washed twice with PBS. After a 12 h of treatment period, the HUVECs were washed, then, the labeled THP‐1 cells (5 × 10^5^ cells per well) were added, and the mixture was incubated at 37 °C for 1 h. Then, the unadhered cells were removed by gentle washing with PBS.^[^ ^67^ ^]^ Images of adherent THP‐1 cells were taken under an inverted fluorescence microscope (Nikon, Japan), and the number of adherent THP‐1 cells was calculated with ImageJ software.

### Statistical Analysis

All the experiments were repeated at least three times to confirm the results. All the quantifications were performed in a blinded fashion. All graphs report the mean ± standard deviation (mean ± SD) values of biological replicates. Two‐way ANOVA followed by Šídák's post hoc test was used for the behavioral data to assess the differences over time. For other data, the log‐rank (Mantel‒Cox) test was used for survival comparisons, Student's *t* test was used for statistical analyses of differences between two groups, and two‐way ANOVA followed by Tukey's test was used for multigroup comparisons. The statistical analysis software used was GraphPad Prism (version 10.0, La Jolla, CA, USA). *p* < 0.05 was considered to indicate statistical significance.

## Conflict of Interest

The authors declare no conflict of interest.

## Author Contributions

W.Z. formulated the concept, designed the studies, and edited the manuscript. M.X. designed the experiments, conducted the experiments, and wrote the paper. W.C. and S.L. analyzed the results. Q.G. revised the manuscript.

## Supporting information



Supporting Information

## Data Availability

The data that support the findings of this study are available from the corresponding author upon reasonable request.
