# Supplementary material for: Annexin A3 Represses Endothelial Permeability and Inflammation During Sepsis via Actin Cytoskeleton Modulation
Source: Adv Sci (Weinh). 2025 Mar 28;12(22):2416904. doi: 10.1002/advs.202416904 (PMC12165021; doi:10.1002/advs.202416904)
Supplement: Supplementary file 1 — Supporting Information [file ADVS-12-2416904-s001.docx]

Supporting Information for

**Annexin A3** **Represses Endothelial Permeability and Inflammation during Sepsis via Actin Cytoskeleton Modulation**

*Manyu Xing, Shuang Liang, Wei Cao, Qulian Guo, Wangyuan Zou**

**This PDF file includes:**

Figure S1 to S8

Table S1 to S3

Key resources table

Supplementary Materials


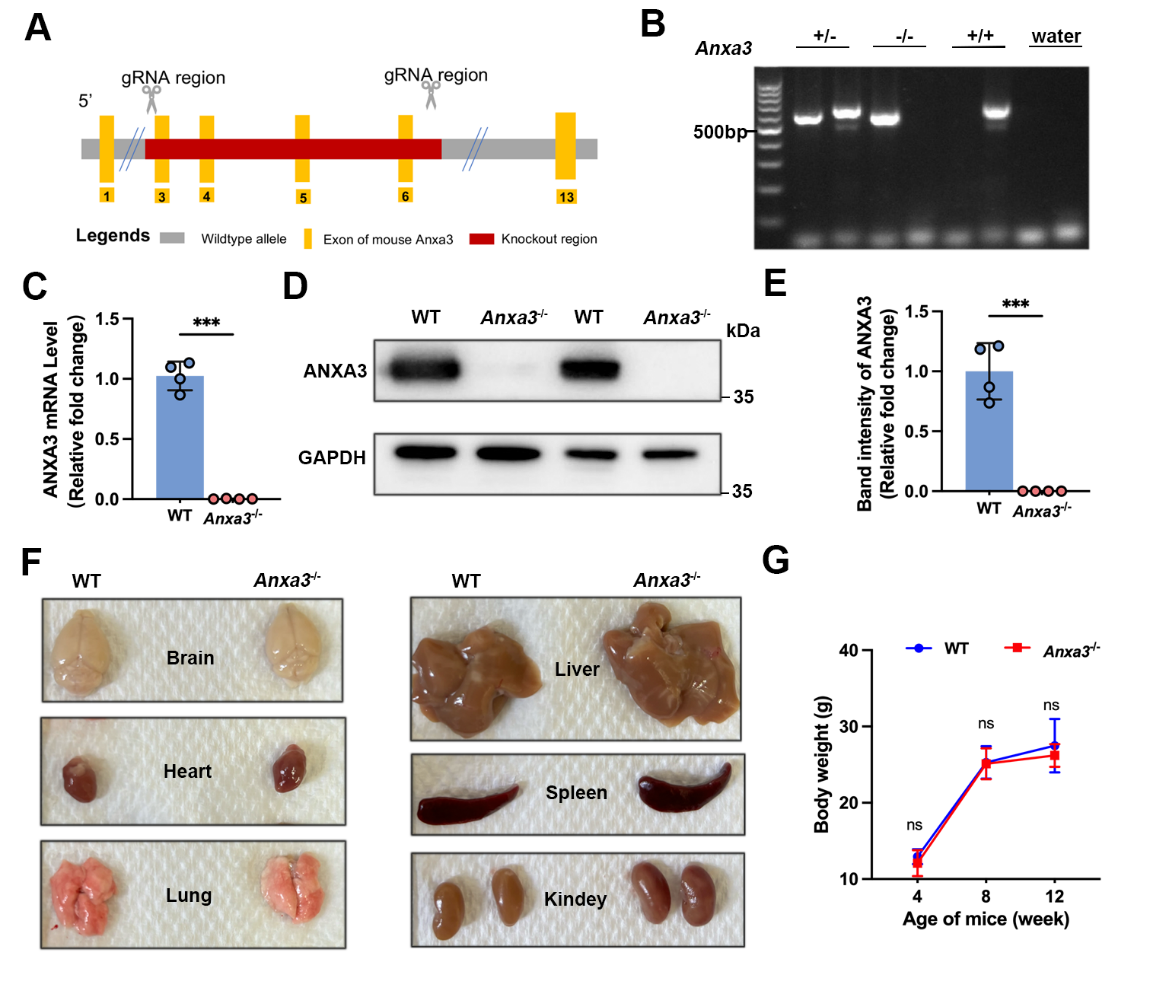


Figure. S1 ANXA3 knockout mice were successfully constructed.

A. The schematic strategy of genome engineering for creating *Anxa*3 knockout mice *Anxa*3^-/-^. To create *Anxa*3 knockout mice by CRISPR/Cas9-mediated genome engineering, gRNA-directed Cas9 endonuclease cleaved the *Anxa*3 gene at the sites indicated on the scheme and made breaks of exon of 3 and 6. Such breaks were repaired by non-homologous end joining and resulted in the disruption of *Anxa*3. (B) Genotyping results of heterozygous (+/-), knockout (-/-) and wild-type (+/+) mice by PCR. (C) ANXA3 mRNA was tested by qPCR in WT or Anxa3-/- mice lung. n = 4, ****P* < 0.001, two-tailed unpaired Student's *t*-test. (D) Western blot showing ANXA3 protein expression in lung from WT or *Anxa*3^-/-^ mice. (E) Densitometric analysis of ANXA3 protein expression. The density of proteins in the WT group was used as a standard (1 arbitrary unit) to compare relative densities in the *Anxa*3^-/-^ group. n = 4, ****P* < 0.001, two-tailed unpaired Student's *t*-test. (F) Gross appearance of brains, hearts, lungs, livers, spleens and kidneys isolated from WT or *Anxa*3^-/-^ mice littermates. (G) Body weight of WT and *Anxa*3^-/-^ littermates at indicated times. n = 9, n.s., no significant difference, two-way ANOVA with Sidak's post hoc test.


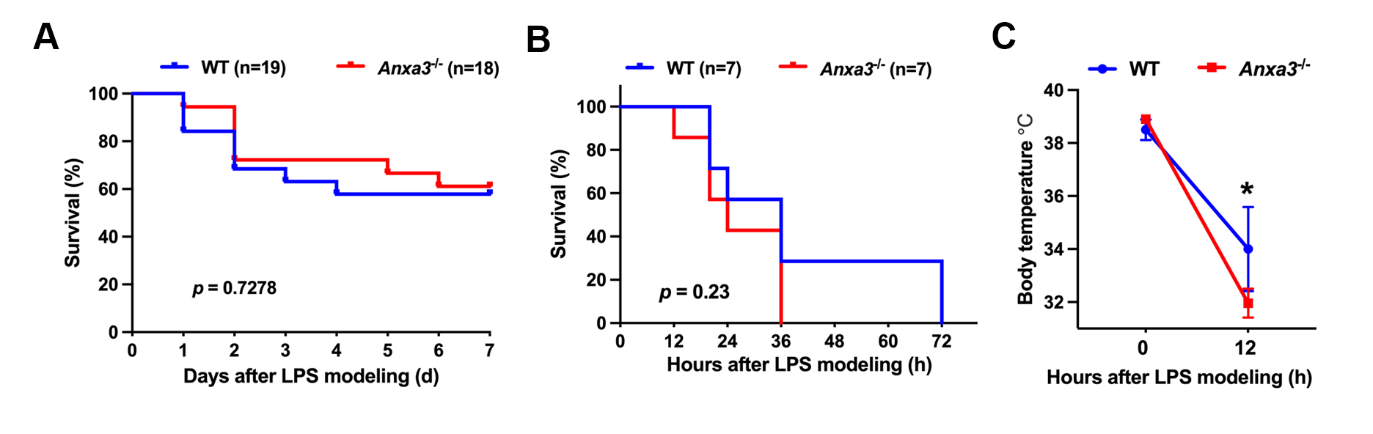


Figure. S2 Gender differences and the dose - effect of the model in *Anxa*3^-/-^ mice after LPS - induced sepsis model.

(A) Survival analysis post-LPS in female WT and *Anxa*3^-/-^ mice, *n* = 18-19/group. *p* = 0.7278, log-rank (Mantel-Cox) test. (B) Survival analysis post-LPS (15 mg/kg) in male WT and *Anxa*3^-/-^ mice, *n* = 7. *p* = 0.031, log-rank (Mantel-Cox) test. (C) At 24 h post-LPS (15 mg/kg), the core body temperature of mice was measured. *n* = 7, **P* < 0.05, two-way ANOVA with Sidak's post hoc test.


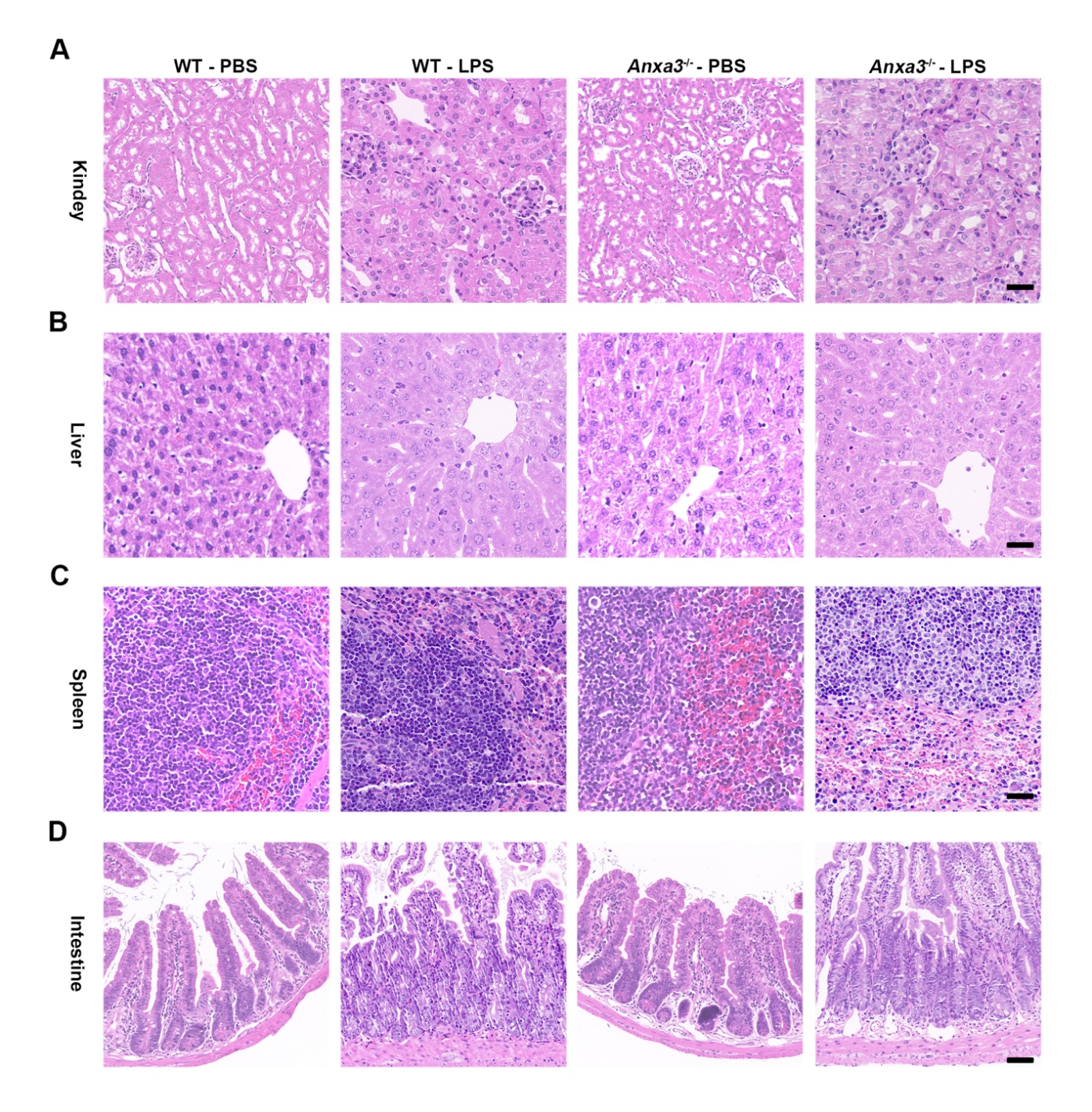


**Figure. S3 Organ injuries in WT and *Anxa*3^-/-^ mice with sepsis.**

At 18 h post-LPS, histological changes in the kidney (A), liver (B), spleen (C), and intestine (D) tissues were assessed with H&E staining from different groups of mice. Scale bar = 25 μm, n = 6.


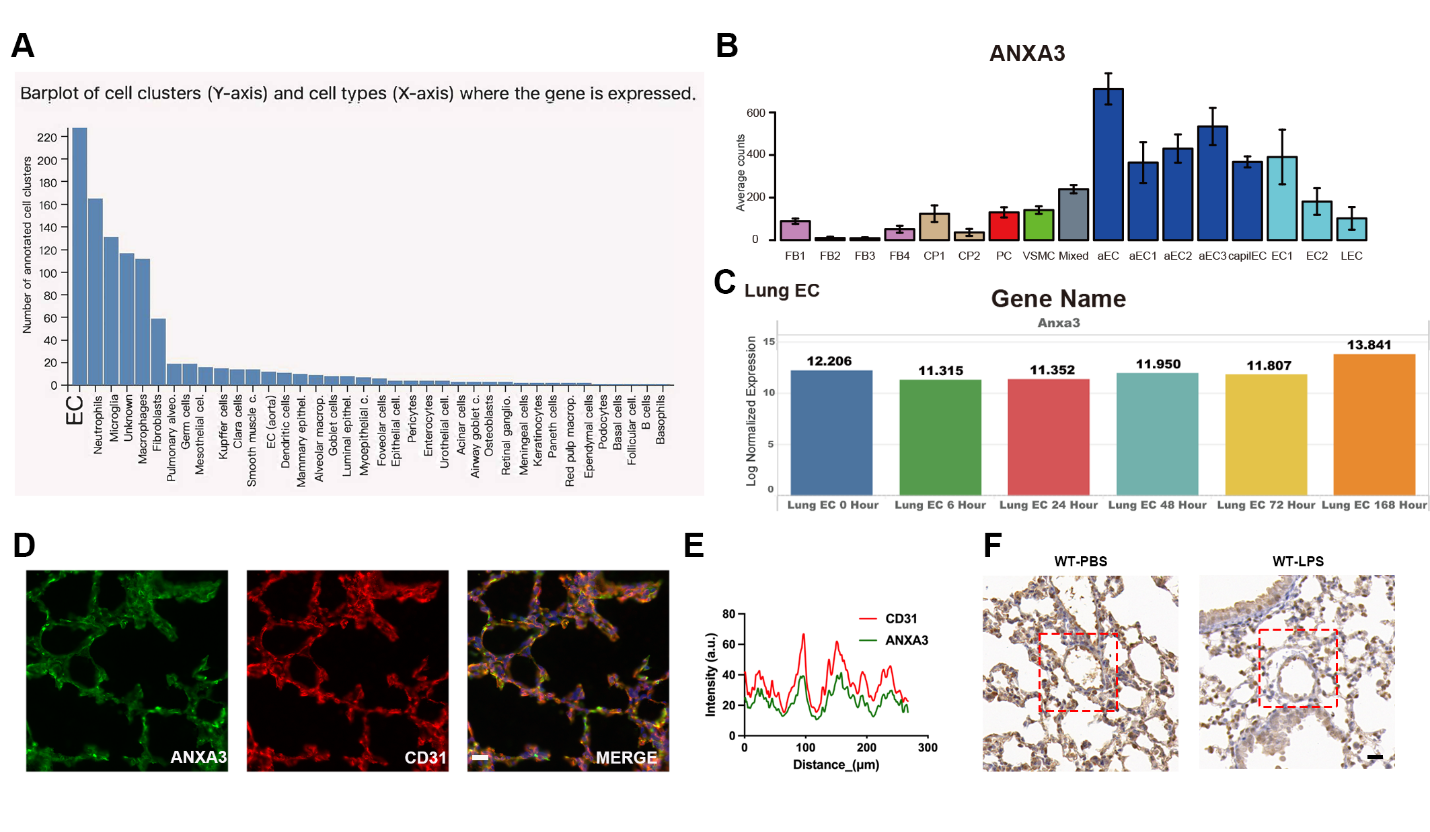
Figure. S4 ANXA3 is highly expressed on endothelial cells.

(A) Barplot of cell clusters (Y-axis) and cell types (X-axis) where the ANXA3 is expressed in mice from scRNA-seq database (<https://panglaodb.se/>) (B) ANXA3 expression in mouse lung vessels was determined by lung vascular scRNA-seq (http://betsholtzlab.org/VascularSingleCells/database.html). FB - Vascular fibroblast-like cells; CP - Cartilage perichondrium; PC - Pericytes; VSMC - Vascular smooth muscle cells; EC - Endothelial cells; capil - capillary; a - arterial; c - continuum; L - Lymphatic; 1,2,3,4 - subtypes. (C) ScRNA-seq data showed the changes of ANXA3 expression in lung endothelial cells at 0, 6, 24-, 48-, 72-, and 168-hour post-LPS administration (http://rehmanlab.org/ribo). (D-E) Immunofluorescence of ANXA3 and CD31 in lung tissues, scale bar = 20 μm. (F) Immunohistochemistry was used to detect ANXA3 expression on lung tissues of wild-type mouse control and LPS modelling 18 h groups; red boxes are perivascular, scale bar = 20 μm.


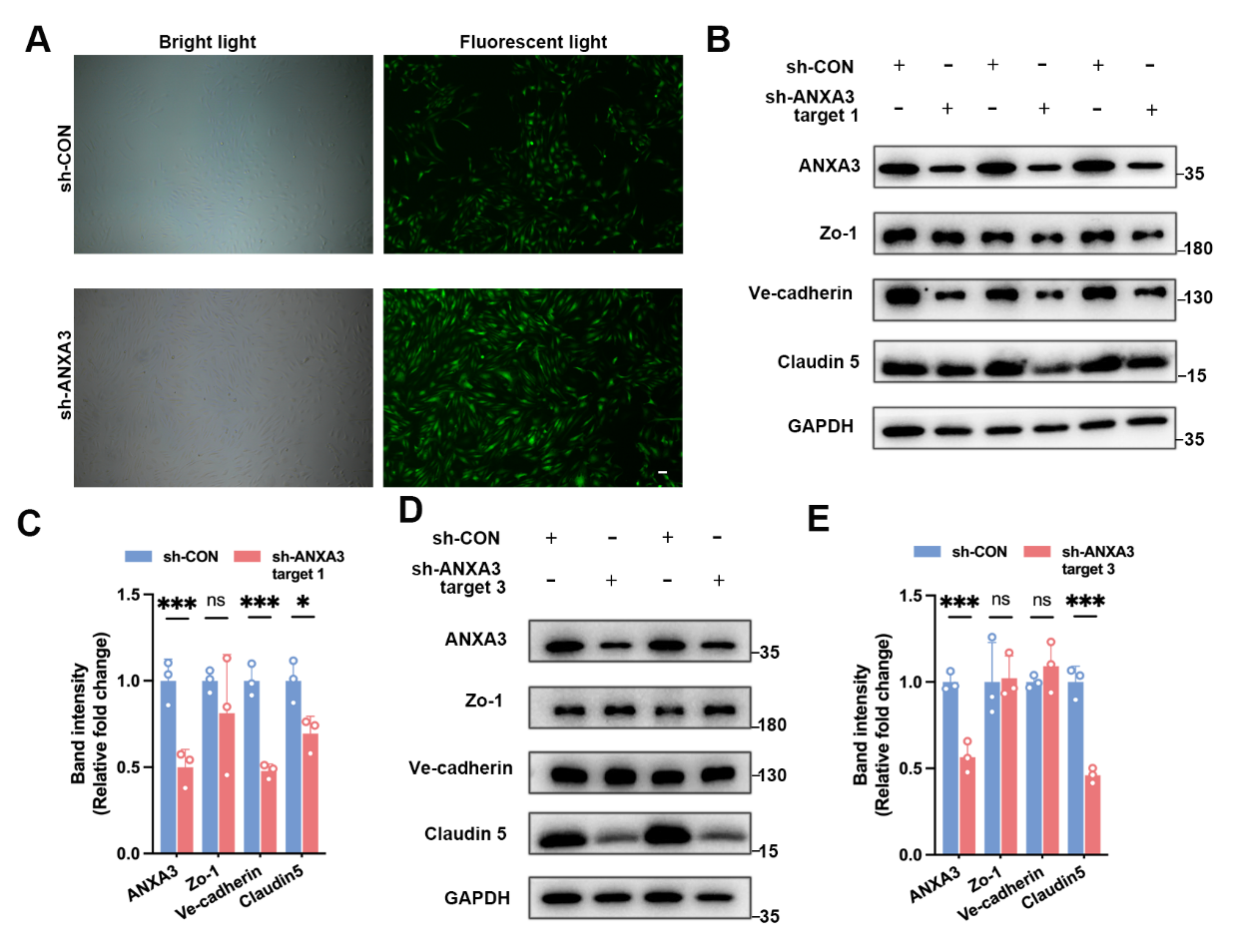


Figure. S5 The degree of downregulation of junction proteins is positively correlated with the degree of ANXA3 knockdown.

(A) HUVECs that were transiently infected by lentivirus with a negative control in MOI = 10 were photographed under bright-field (left) and fluorescent (right) conditions. Scale bar = 100 μm. (B-C) Gel images and quantification of western blots for ANXA3, ZO-1, VE-cadherin, and claudin-5 expressions in HUVECs transiently infected by lentivirus with negative control sequence and shRNA-1 sequence. n = 3, **P* < 0.05, ***P* < 0.01, ****P* < 0.001, two-tailed unpaired Student's *t*-test. (D-E) Gel images and quantification of western blots for ANXA3, ZO-1, VE-cadherin, and claudin-5 expressions in HUVECs transiently infected by lentivirus with negative control sequence and shRNA-3 sequence. n = 3, ****P* < 0.001, two-tailed unpaired Student's *t*-test.


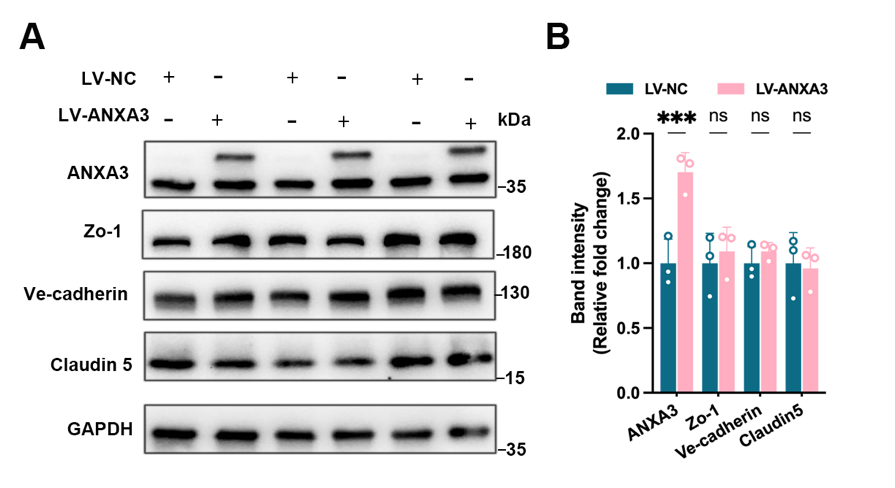


**Figure. S6 Overexpression of ANXA3 did not enhance the expression of junction proteins.**

(A-B) Gel images and quantification of western blots for ANXA3, ZO-1, VE-cadherin, and claudin-5 expressions in HUVECs infected by lentivirus with negative control sequence and ANXA3 overexpression sequence. n = 3, ****P* < 0.001, two-tailed unpaired Student's *t*-test.


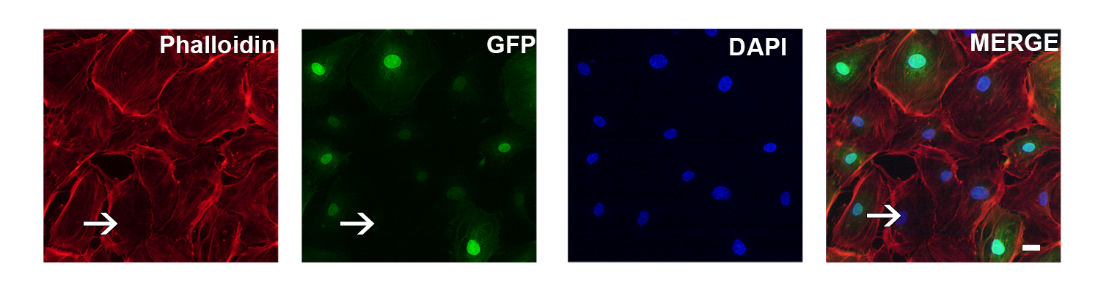


**Figure. S7 The synthesis of Rhodamine-phalloidin staining and the lentivirus infection (GFP) in Figure 3 G.** The arrow indicates the cell that has not been infected with the shRNA for knocking down ANXA3. Scale bar = 20 µm.


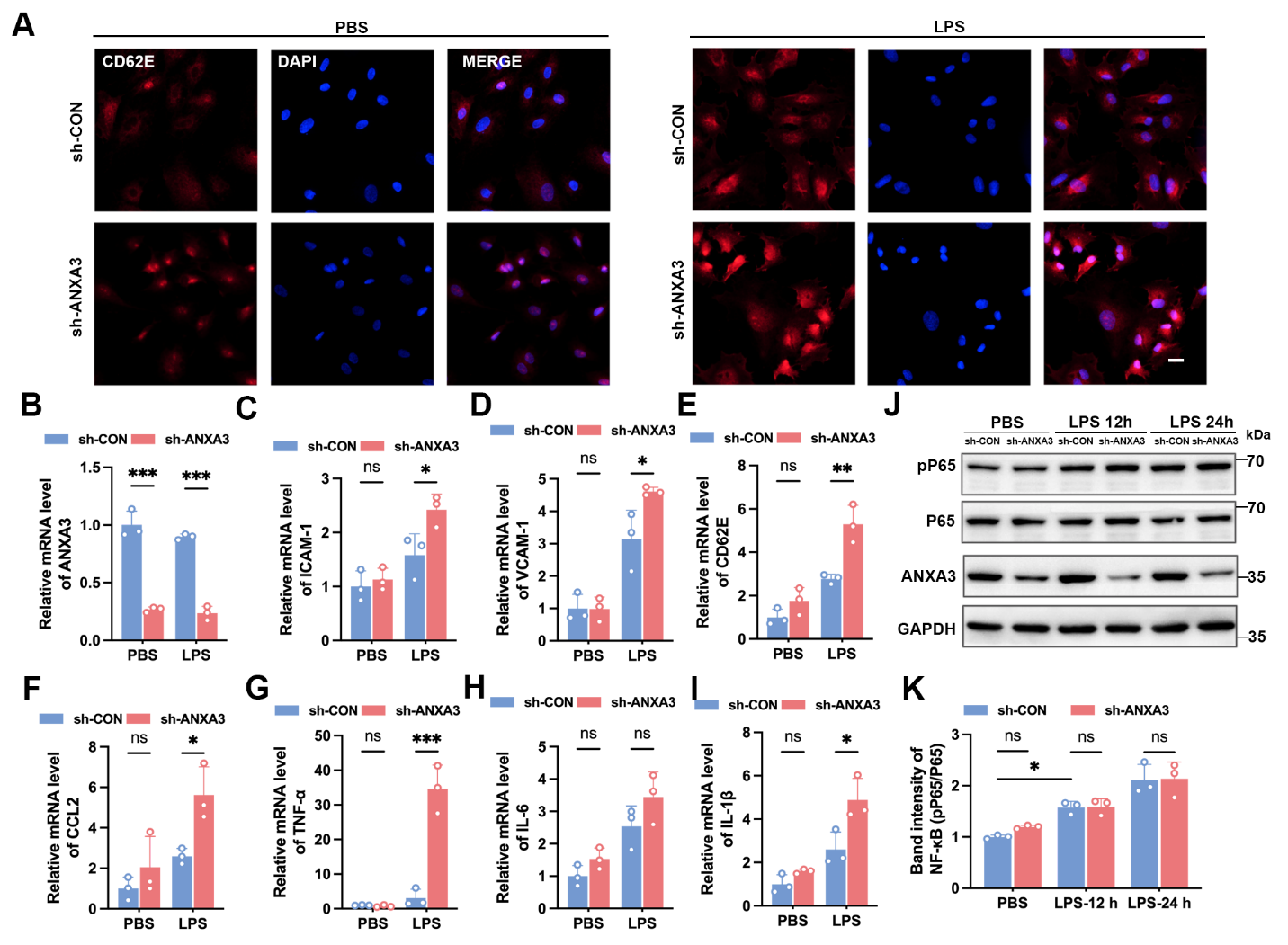


**Figure. S8** **ANXA3 limits LPS-induced endothelial inflammation in HUVECs independent of NF - κB activation.**

(A) The control HUVECs (sh-CON) and the HUVECs with knocked-down ANXA3 (sh-ANXA3) were stimulated by LPS. The position and level of CD62E were detected by immunofluorescence. Scale bar = 10 μm. (B-I) The mRNA level of ANXA3, adhesion molecules (CD62E, ICAM - 1, VCAM - 1), chemokine CCL2, and inflammatory cytokines (TNF - α, IL - 1β, IL6) in sh-CON and sh-ANXA3 after LPS stimulation were detected by qPCR. n = 3, **P* < 0.05, ***P* < 0.01, ****P* < 0.001. Two-way ANOVA followed by Tukey’s test was used for multiple-group comparisons. (J-K) The phosphorylation of P65 was detected by WB in sh-CON and sh-ANXA3 after LPS stimulation. n = 3, **P* < 0.05. Two-way ANOVA followed by Tukey’s test was used for multiple-group comparisons.

Table S1. The target sequences of shRNA to knock down the human ANXA3 expression.

| shRNA | Target Seq（5’-3’） |
| --- | --- |
| Target-1 | CACCAGCAGTCTTTGATGCAA |
| Target-2 | GGGCACGGATGAAGACAAATT |
| Target-3 | GAGCCTTGAAGGGTATTGGAA |

Table S2. Primer sequences for mouse genes

| Gene | Forward（5’-3’） | Reverse（5’-3’） |
| --- | --- | --- |
| *Anxa3* | CAAATTCACCGAGATCCTGT | TGCTGGAGTGCTGTACGAAA |
| *Gapdh* | GGTGAAGGTCGGTGTGAACG | CTCGCTCCTGGAAGATGGTG |
| *Icam-1* | GTGATGCTCAGGTATCCATCCA | CACAGTTCTCAAAGCACAGCG |
| *Vcam-1* | GTTCCAGCGAGGGTCTACC | AACTCTTGGCAAACATTAGGTGT |
| *Cd62E* | ATGCCTCGCGCTTTCTCTC | GTAGTCCCGCTGACAGTATGC |
| *Ccl2* | TTAAAAACCTGGATCGGAACCAA | GCATTAGCTTCAGATTTACGGGT |

Table S3. Primer sequences for human genes

| Gene | Forward（5’-3’） | Reverse（5’-3’） |
| --- | --- | --- |
| *ANXA3* | GAGAGGTCAAATGCACAGCG | CTGGTGGAGTCACTAGGGC |
| *GAPDH* | ACAACTTTGGTATCGTGGAAGG | GCCATCACGCCACAGTTTC |
| *TNF-α* | GAGGCCAAGCCCTGGTATG | CGGGCCGATTGATCTCAGC |
| *IL-6* | ACTCACCTCTTCAGAACGAATTG | CCATCTTTGGAAGGTTCAGGTTG |
| *IL-1β* | ATGATGGCTTATTACAGTGGCAA | GTCGGAGATTCGTAGCTGGA |
| *ICAM-1* | ATGCCCAGACATCTGTGTCC | GGGGTCTCTATGCCCAACAA |
| *VCAM-1* | TTTGACAGGCTGGAGATAGACT | TCAATGTGTAATTTAGCTCGGCA |
| *CD62E* | CAGCAAAGGTACACACACCTG | CAGACCCACACATTGTTGACTT |
| *CCL2* | CAGCCAGATGCAATCAATGCC | TGGAATCCTGAACCCACTTCT |

**Key resources table**

| REAGENT or RESOURCE | SOURCE | IDENTIFIER |
| --- | --- | --- |
| Antibodies | | |
| ANXA3 | Sigma-aldrich | HPA013398 |
| Cd31 | R&D | AF3628 |
| GAPDH | Bioworld | BS72410 |
| Ve-cadherin | Santa cruz | sc-9989 |
| Zo-1 | Introvigen | 33-9100 |
| Claudin 5 | Introvigen | 34-1600 |
| Flag | Biodragon | B1001 |
| β-actin | Bioworld | AP0731 |
| ICAM-1 | Santa cruz | sc-8439 |
| CD62E | Proteintech | 20849-1-ap |
| NF-κB p65 | Abcam | ab16502 |
| NF-κB pp65 | Cell Signaling Technology | 3033 |
| pATF2 | Santa cruz | sc-8398 |
| ATF2 | Abcam | ab32061 |
| HRP-conjugated Goat Anti-Rabbit IgG | Proteintech | SA00001-2 |
| HRP-conjugated Goat Anti-Mouse IgG | Proteintech | SA00001-1 |
| Goat Anti-Rabbit IgG H&L (Alexa Fluor® 488) | Abcam | ab150077 |
| Goat Anti-Mouse IgG H&L (Alexa Fluor® 488) | Abcam | ab150113 |
| Alexa Fluor 594-conjugated Goat Anti-mouse IgG | Jackson ImmunoResearch | AB2338871 |
| Alexa Fluor 594-conjugated Goat Anti-Rabbit IgG | Jackson ImmunoResearch | AB2338059 |
| Bacterial and virus strains | | |
| GV492 | Genechem | n/a |
| GV493 | Genechem | n/a |
| Biological samples |  |  |
| n/a |  |  |
| Chemicals, peptides, and recombinant proteins | | |
| Evans blue | Sigma | E2129 |
| FITC-dextran | Sigma | 46945 |
| LPS-EB (LPS from *E*. *coli* 011:B4) | Sigma | L2630 |
| Dil | KeyGEN biotech | KGE2601-10 |
| Cytochalasin D | MedChemExpress | HY-N6682 |
| SP600125 | MedChemExpress | HY-12041 |
| Deposited data | | |
| n/a |  |  |
| Experimental models: Cell lines | | |
| THP-1 cell line | Hysigen Bioscience | TCH-C361 |
| HUVECs | National stem cell translational resource center | DFSC-EC-01 |
| Experimental models: Organisms/strains | | |
| Mouse: C57BL/6Ncr | Cyagen Biosciences | n/a |
| Mouse: *Anxa3*^-/-^ | Cyagen Biosciences | n/a |
| Recombinant DNA | | |
| Flag-ANXA3 | Genechem |  |
| Software and algorithms | | |
| Prism 10 | Graphpad software | Version 10 |
| Image J software |  | Version 2.3.0 |
| Other | | |
| n/a |  |  |
